# Efficient augmentation and relaxation learning for individualized treatment rules using observational data

**Published:** 2019

**Authors:** Ying-Qi Zhao, Eric B. Laber, Yang Ning, Sumona Saha, Bruce E. Sands

**Affiliations:** Public Health Sciences Division, Fred Hutchinson Cancer Research Center, Seattle, WA, 98109, USA; Department of Statistics, North Carolina State University, Raleigh, NC, 27695, USA; Department of Statistical Science, Cornell University, Ithaca, NY, 14853, USA; School of Medicine and Public Health, University of Wisconsin, Madison, WI, 53705, USA; Division of Gastroenterology, Icahn School of Medicine at Mount Sinai, New York, NY, 10029, USA

**Keywords:** Individualized treatment rules, convex surrogate, double-robustness, classification, personalized medicine

## Abstract

Individualized treatment rules aim to identify if, when, which, and to whom treatment should be applied. A globally aging population, rising healthcare costs, and increased access to patient-level data have created an urgent need for high-quality estimators of individualized treatment rules that can be applied to observational data. A recent and promising line of research for estimating individualized treatment rules recasts the problem of estimating an optimal treatment rule as a weighted classification problem. We consider a class of estimators for optimal treatment rules that are analogous to convex large-margin classifiers. The proposed class applies to observational data and is doubly-robust in the sense that correct specification of either a propensity or outcome model leads to consistent estimation of the optimal individualized treatment rule. Using techniques from semiparametric efficiency theory, we derive rates of convergence for the proposed estimators and use these rates to characterize the bias-variance trade-off for estimating individualized treatment rules with classification-based methods. Simulation experiments informed by these results demonstrate that it is possible to construct new estimators within the proposed framework that significantly outperform existing ones. We illustrate the proposed methods using data from a labor training program and a study of inflammatory bowel syndrome.

## 1. Introduction

There is a growing consensus that the best possible care results from treatment decisions that are carefully tailored to individual patient characteristics (Sox and Greenfield, 2009). Individualized treatment rules (ITRs) formalize tailored treatment decisions as a function from patient information to a recommended treatment. We define an optimal ITR as maximizing the mean of a pre-specified clinical outcome if applied to recommend treatments in a population of interest (see Linn et al., 2016, for alternative definitions of optimality). With expanding access to patient-level data through electronic health records, adverse event reporting, insurance claims, and billing records, there is increasing interest in estimating optimal ITRs from observational data. An important use of an estimated optimal ITR is hypothesis-generation whereby the estimated optimal rule is used to discover covariate-treatment interactions or identify subgroups of patients with large treatment effects. In such applications, it is useful to directly control the class of ITRs within which the optimal ITR will be estimated. The form of this class can be chosen to ensure interpretability, enforce logistical or cost constraints, or make the tests of certain clinical hypotheses overt.

One approach to estimating an optimal ITR is to model some or all of the conditional distribution of the outcome given treatments and covariates and then to use this estimated distribution to infer the optimal ITR. These approaches are sometimes called indirect methods as they indirectly specify the form of the optimal ITR through postulated models for components of the conditional outcome distribution. Indirect methods have dominated the literature on estimating optimal ITRs; examples of indirect estimation methods include variations of *g*-estimation in structural nested models (Robins, 1989, 1997; Murphy, 2003; Robins, 2004); *Q*- and *A*-learning (Zhao et al., 2009; Qian and Murphy, 2011; Moodie et al., 2012; Chakraborty and Moodie, 2013; Schulte et al., 2014), and regret regression (Henderson et al., 2009). However, a major drawback with these approaches is that the postulated outcome models dictates the class of possible ITRs. A consequence is that to obtain a simple ITR requires specification of simple outcome models, which may not be correctly specified. Moreover, if these outcome models are misspecified, the foregoing methods may not be consistent for the optimal ITR within the class implied by the outcome models. For example, to ensure a linear ITR using *Q*-learning, it is common to use a linear conditional mean model. It can be shown that if the linear mean model is misspecified then the estimated optimal ITR using *Q*-learning need not converge to the optimal linear ITR (Qian and Murphy, 2011). Alternatively, flexible outcome models that mitigate the risk of misspecification (e.g., Zhao et al., 2009; Qian and Murphy, 2011; Moodie et al., 2013) can induce a class of ITRs that is difficult or impossible to interpret (see Section 2 for details).

An alternative to indirect estimation is to decouple models for the conditional outcome distribution from the class of ITRs. One way to do this is to form a flexible estimator of the mean outcome as a function of the ITR that is consistent under a large class of potential generative models and then to use the maximizer of this function over a pre-specified class of ITRs as the estimator of the optimal ITR. These approaches are called direct (Laber et al., 2014), policy-search (Sutton and Barto, 1998; Szepesvári, 2010), policy learning (Athey and Wager, 2017) or value-search (Davidian et al., 2014) estimators. An advantage of direct estimators is that they permit flexible, e.g., semi- or non-parametric, models for modeled portions of the outcome distribution yet still control the form of the estimated optimal ITR. Direct estimators include outcome weighted learning (Zhao et al., 2012, 2015a, 2015b), robust value-search estimators (Zhang et al., 2012a, 2012b, 2013); marginal structural mean models (Robins et al., 2008; Orellana et al., 2010); and Q-learning with policy-search (Taylor et al., 2015; Zhang et al., 2015, 2017).

While the foregoing methods represent significant progress in direct estimation, computational and theoretical gaps remain. Outcome weighted learning uses a convex relaxation of an inverse-probability weighted estimator (IPWE) of the mean outcome. This convex relaxation makes their method computationally efficient and scalable to large problems; in addition, convexity simplifies derivations of convergence rates and generalization error bounds. However, the IPWE is known to be unstable under certain generative models (Zhang et al., 2012a, 2012b), and theoretical guarantees for outcome weighted learning were developed only for data from a randomized clinical trial. Robust value-search estimators directly maximize an augmented IPWE (AIPWE). The AIPWE is semi-parametric efficient and is significantly more stable than the IPWE. However, the AIPWE is a discontinuous function of the observed data, which makes direct maximization computationally burdensome even in moderate sized problems and complicates theoretical study of these estimators. We establish the theory for both AIPW and its convex relaxation, which fills the gap in the current literature on direct search methods. Marginal structural mean models are best suited for problems where the ITR depends only on a very small number of covariates. Liu et al. (2016) proposed a robust method for estimating optimal treatment rules in a multi-stage setup. At each stage in a multi-stage setup, they proposed a robust weight to replace the original weight in OWL based on the idea of augmentation. However, they still require consistent estimation of the propensity score at the present stage. In particular, their proposal for the single stage problem still relies on an IPWE, and does not possess the double robustness property.

We propose a class of estimators representable as the maximizer of a convex relaxation of the AIPWE; we term this class of estimators Efficient Augmentation and Relaxation Learning (EARL). EARL is computationally efficient, theoretically tractable, and applies to both observational and experimental data. Furthermore, EARL contains outcome weighted learning (OWL) (Zhao et al., 2012) as a special case. However, EARL is considerably more general than OWL, and this generality leads to new insights about classification-based estimation of ITRs, new algorithms, and new theoretical results. Unlike OWL, EARL makes use of both a propensity score and an outcome regression model. Estimators within the EARL framework are doubly-robust in the sense that they consistently estimate the optimal ITR if either the propensity score model or outcome regression model is correctly specified. Within the EARL framework, we are able to characterize convergence rates across a range of convex relaxations, propensity score models, and outcome regression models. In particular, making use of sample splitting, we are able to remove the dependence in estimating the nuisance functions and in constructing the estimated ITR. We show that under all convex relaxations considered, a fast convergence rate of the estimated optimal ITR can be achieved, and that the estimation of the propensity score and outcome regression models need not affect the upper bound of this rate. Our theoretical results complement existing work on convergence rate for estimating optimal treatment decision rules, which primarily compared the estimated rules to the best-in-class rule (Kitagawa and Tetenov, 2017; Athey and Wager, 2017; ?). The proposed method has been implemented in R and is freely available through the ‘DynTxRegime’ package hosted on the comprehensive R network (cran.org).

In Section 2, we introduce the EARL class of estimators. In Section 3, we investigate the theoretical properties of estimators within this class. In Section 4, we use simulation experiments to investigate the finite sample performance of EARL estimators. In Section 5, we present illustrative case studies using data from a labor training program and an inflammatory bowel disease study. In Section 6, we make concluding remarks and discuss potential extensions.

## 2. Methods

In this section, we first provide background of the proposed method. We then introduce Efficient Augmentation and Relaxation Learning (EARL) in details.

### 2.1 Background and preliminaries

The observed data, ${\left\{\left(X_{i},A_{i},Y_{i}\right)\right\}}_{i=1}^{n}$, comprise *n* independent, identically distributed copies of (***X***, *A, Y* ), where: ***X*** ∈ ℝ^*p*^ denotes baseline subject measurements; *A* ∈ {−1, 1} denotes the assigned treatment; and *Y* ∈ ℝ denotes the outcome, coded so that higher values are better. In this context, an ITR, *d*, is a map from ℝ^*p*^ into {−1, 1} so that a patient presenting with ***X*** = ***x*** is recommended treatment *d*(***x***). Let D denote a class of ITRs of interest. To define the optimal ITR, denoted d*, we use the framework of potential outcomes (Rubin, 1974; Splawa-Neyman et al., 1990). Let *Y* (*a*) denote the potential outcome under treatment *a* ∈ {−1, 1} and define $Y(d)=\sum_{a\in \{-1,1\}}Y(a)I\{a=d(X)\}$ to be the potential outcome under *d*. The marginal mean outcome V(d)≜E{Y(d)} is called the value of the ITR *d*. The optimal ITR satisfies d*∈D and *V* (d*) ≥ *V* (*d*) for all d∈D. Note that this definition of optimality depends on the class D. To express the value in terms of the data generating model, we assume: (i) strong ignorability, {*Y*(−1), *Y*(1)} ⫫ *A*|***X*** (Rubin, 1974; Robins, 1986; Splawa-Neyman et al., 1990); (ii) consistency, *Y* = *Y* (*A*); and (iii) positivity, there exists *τ* > 0 so that *τ* < *P*(*A* = *a*|***X***) for each *a* ∈ {−1, 1} with probability one. These assumptions are common and well-studied (see Schulte et al., 2014, for a recent review of potential outcomes for treatment rules). Assumption (i) is true in a randomized study but unverifiable in an observational study (Bang and Robins, 2005).

Define Q(x,a)≜E(Y|X=x,A=a), then under the foregoing assumptions, it can be shown that

(1) V(d)=E[Q{X,d(X)}],

from which it follows that $d^{*}(x)=\arg\;\max_{a\in \{-1,1\}}Q(x,a)$. Q-learning is a common regression-based indirect approach for estimating d* wherein an estimator $\hat{Q}(x,a)$ of *Q*(***x***, *a*) is constructed and subsequently the estimated optimal rule is $\hat{d}(x)=\arg\;\max_{a}\hat{Q}(x,a)$. Let Q denote the postulated class of models for *Q*(***x***, *a*), then the set of possible decision rules obtained using *Q*-learning is $D=\left\{d:d(x)=\arg\max_{a}Q(x,a),Q\in Q\right\}$. Thus, there is an inherent trade-off between choosing Q to be sufficiently rich to reduce the risk of model misspecification and the resultant complexity of the resultant class of ITRs.

Direct estimators specify a class of candidate ITRs independently from postulated models for some or all of the generative model. Let D denote a class of ITRS; direct search estimators first construct an estimator of the value function, $\text{say}\;\hat{V}(\cdot )$, and then choose $\hat{d}=\arg\max_{d\in D}\hat{V}(d)$ as the estimator of d*. Thus, a complex model space for *V* (·) need not imply a complex class of rules D. However, the class of models for *V* (·) must be sufficiently rich to avoid implicit, unintended restrictions on $\hat{d}$. To avoid such restrictions and to avoid model-misspecification, it is common to use a flexible class of semi- or non-parametric models for *V* (·).

### 2.2 Augmentation for the value function

Define the propensity score π(a;x)≜P(A=a|X=x), then

(2) $$V(d)=E\left[\frac{Y}{\pi (A;X)}I\{A=d(X)\}\right],$$

where *I*{·} denotes the indicator function (e.g., Qian and Murphy, 2011). Unlike (1), the preceding expression does not require an estimator of the *Q*-function. Given an estimator of the propensity score, $\hat{\pi }(a;x)$, a plug-in estimator for *V* (*d*) based on (2) is the inverse probability weighted estimator $\left(\mathrm{IPWE})\;{\hat{V}}^{\text{IPWE}}(d)≜{\mathbb{P}}_{n}[YI\{A=d(X)\}/\hat{\pi }(A;X)\right]$, where ${\mathbb{P}}_{n}$ is the empirical distribution. The IPWE has potentially high variance as it only uses outcomes from subjects whose treatment assignments coincide with those recommended by *d*.

One approach to reduce variability is to augment the IPWE with a term involving both the propensity score and the *Q*-function that is estimated using data from all of the observed subjects (Robins et al., 1994; Cao et al., 2009). Let $\hat{Q}(x,a)$ denote an estimator of *Q*(***x***, *a*). The augmented inverse probability weighted estimator is

(3) $${\hat{V}}^{\text{AIPWE}}(d)≜{\mathbb{P}}_{n}\left[\frac{YI\{A=d(X)\}}{\hat{\pi }\{d(X);X\}}-\frac{I\{A=d(X)\}-\hat{\pi }\{d(X);X\}}{\hat{\pi }\{d(X);X\}}\hat{Q}\{X,d(X)\}\right].$$

It can be seen that ${\hat{V}}^{\text{AIPWE}}(d)$ is equal to ${\hat{V}}^{\text{IPWE}}(d)$ plus an estimator of zero built using outcomes from all subjects regardless of whether or not their treatment assignment is consistent with *d*. If $\hat{Q}(x,a)\equiv 0$ then ${\hat{V}}^{\mathrm{AIPWE}}(d)={\hat{V}}^{\mathrm{IPWE}}(d)$ for all *d*.

Hereafter, we use $\hat{Q}(x,a)$ and $\hat{\pi }(a;x)$ to denote generic estimators of the *Q*-function and propensity score. The following assumption is used to establish double robustness of ${\hat{V}}^{\text{AIPWE}}(d)$.

#### Assumption 1

$\hat{Q}(x,a)$ *and* $\hat{\pi }(a;x)$ *converge in probability uniformly to deterministic limits Q*^*m*^(***x***, *a*) *and π*^*m*^(*a*; ***x***).

This assumption does not require that the estimators $\hat{Q}(x,a)$, $\hat{\pi }(a;x)$ are consistent for the truth, only that they converge to fixed functions. The following result is proved in Web Appendix A.

#### Lemma 2.1

*Let* d∈D *be fixed. If* either *π*^*m*^(*a*; ***x***) = *π*(*a*; ***x***) *or Q*^*m*^(***x***, *a*) = *Q*(***x***, *a*) *for all* (***x***, *a*) *outside of a set of measure zero, then* ${\hat{V}}^{AIPWE}(d)\to_{p}V^{AIPWE,m}(d)=V(d)$, *where*

$$V^{AIPWE,m}(d)≜E\left[\frac{YI\{A=d(X)\}}{\pi^{m}(A;X)}-\frac{I\{A=d(X)\}-\pi^{m}\{d(X);X\}}{\pi^{m}\{d(X);X\}}Q^{m}\{X,d(X)\}\right].$$

The preceding result shows that ${\hat{V}}^{AIPWE}(d)$ is doubly-robust in the sense that if either the propensity model or the modeled *Q*-function is consistent, but not necessarily both, then ${\hat{V}}^{AIPWE}(d)$ is consistent for *V* (*d*). Thus, the maximizer of ${\hat{V}}^{AIPWE}(d)$ over d∈D is termed a doubly-robust estimator of the optimal treatment rule (Zhang et al., 2012a, 2012b, 2013). However, because ${\hat{V}}^{AIPWE}(d)$ is not continuous, computing this doubly-robust estimator can be computationally infeasible even in moderate problems (Zhang et al., 2012a). Instead, we form an estimator by maximizing a concave relaxation of ${\hat{V}}^{AIPWE}(d)$. Maximizing this concave relaxation is computationally efficient even in very high-dimensional problems. We show that the maximizer of this relaxed criteria remains doubly-robust. Furthermore, we show that the rates of convergence of the proposed estimators depend on the chosen concave relaxation, the chosen propensity model, and the chosen model for the *Q*-function. The relationships among these choices provides new knowledge about direct search estimators based on concave surrogates (Zhang et al., 2012; Zhao et al., 2012, 2015a, 2015b).

### 2.3 Efficient augmentation and relaxation learning (EARL)

Let M be the class of measurable functions from ℝ^*p*^ into ℝ. Any decision rule *d*(***x***) can be written as *d*(***x***) = sgn{*f*(***x***)} for some function f∈M, where we define sgn(0) = 1. For *d*(***x***) = sgn{*f*(***x***)}, *I*{*a* = *d*(***x***)} = *I*{*af*(***x***) ≥ 0}. Define *V* (*f*), *V*^IPWE,*m*^(*f*), and *V* ^AIPWE,*m*^(*f*) by substituting *I*{*Af*(***X***) ≥ 0} for *I*{*A* = *d*(***X***)} in their respective definitions. Define

$$W_{a}^{m}=W_{a}\left(Y,X,A,\pi^{m},Q^{m}\right)=\frac{YI(A=a)}{\pi^{m}(a;X)}-\frac{I(A=a)-\pi^{m}(a;X)}{\pi^{m}(a;X)}Q^{m}(X,a),a\in \{-1,1\}.$$

The following result shows that maximizing ${\hat{V}}^{\text{AIPWE}}(f)$ is equivalent to minimizing a sum of weighted misclassification rates; a proof is given in Web Appendix B.

#### Lemma 2.2

*Assume that P*{*f*(***X***) = 0} = 0. *Define* ${\hat{f}}_{n}=\arg\sup_{f\in M}{\hat{V}}^{\text{AIPWE}}(f)$, *then*

$${\hat{f}}_{n}=\arg\;\underset{f\in M}{\inf}\;{\mathbb{P}}_{n}\left[\left|{\hat{W}}_{1}\right|I\left\{\mathrm{sgn}\left({\hat{W}}_{1}\right)f(X)<0\right\}+\left|{\hat{W}}_{-1}\right|I\left\{-\mathrm{sgn}\left({\hat{W}}_{-1}\right)f(X)<0\right\}\right],$$

*where* ${\hat{W}}_{a}=W_{a}(Y,X,A,\hat{\pi },\hat{Q})$, *a* = ∈ {−1, 1}.

Lemma 2.2 shows that the estimator, ${\hat{f}}_{n}$, which maximizes ${\hat{V}}^{\text{AIPWE}}(f)$ over f∈M, can be viewed as minimizing a sum of weighted 0–1 losses. In this view, the class labels are $\mathrm{sgn}\left({\hat{W}}_{a}\right)\cdot a$ and the misclassification weights are $\left|{\hat{W}}_{a}\right|$, *a* = ∈ {−1, 1} (see Zhang et al., 2012b, 2013). Directly minimizing the combined weighted 0–1 loss is a difficult non-convex optimization problem (Laber and Murphy, 2011). One strategy to reduce computational complexity is to replace the indicator function with a convex surrogate and to minimize the resulting relaxed objective function (Freund and Schapire, 1999; Bartlett et al., 2006; Hastie et al., 2009). This strategy has proved successful empirically and theoretically in classification and estimation of optimal treatment rules (Zhao et al., 2012). However, unlike previous applications of convex relaxations to the estimation of optimal treatment rules, we establish rates of convergence as a function of the: (i) choice of convex surrogate; (ii) convergence rate of the postulated propensity score estimator; and (iii) convergence rate the postulated *Q*-function estimator. We characterize the relationship among these three components in Section 3.

The function *f* is conceptualized as being a smooth function of ***x*** that is more easily constrained to possess certain desired structure, e.g., sparsity, linearity, etc. Thus, we will focus on estimation of *f* within a class of functions F called the approximation space; we assume that F is a Hilbert space with norm ‖ · ‖k. Let *ϕ*: ℝ → ℝ denote a convex function and define EARL estimators as those taking the form

(4) $${\tilde{f}}_{n}^{\lambda_{n}}=\arg\;\underset{f\in F}{\inf}\;{\mathbb{P}}_{n}\left[\left|{\hat{W}}_{1}\right|\phi \left\{\mathrm{sgn}\left({\hat{W}}_{1}\right)f(X)\right\}+\left|{\hat{W}}_{-1}\right|\phi \left\{-\mathrm{sgn}\left({\hat{W}}_{-1}\right)f(X)\right\}\right]+\lambda_{n}\|f{\|}^{2},$$

where *λ*_*n*_‖*f*‖^2^ is included to reduce overfitting and *λ*_*n*_ ≥ 0 is a (possibly data-dependent) tuning parameter. Throughout, we assume that *ϕ*(*t*) is one of the following: hinge loss, *ϕ*(*t*) = max(1 – *t,*0); exponential loss, *ϕ*(*t*) = *e*^−*t*^; logistic loss, *ϕ*(*t*) = log(1 + *e*^−*t*^); or squared hinge loss, *ϕ*(*t*) = {max(1 – *t,* 0)}^2^. However, other convex loss functions are possible provided that they are differentiable, monotone, strictly convex, and satisfy (0) = 1 (Bartlett et al., 2006). As noted previously, Zhao et al. (2012) proposed a special case of EARL called outcome weighted learning, which set (*t*) = max(0, 1 – *t*), $\hat{Q}(x,a)\equiv 0$, and assumed that the propensity score was known. Thus, as noted previously, EARL is considerably more general than OWL and, as shown in Section 4, the choice of a non-null model for the *Q*-function and alternative surrogate loss functions can lead to dramatically improved finite sample performance.

### 2.4 EARL via sample splitting

To facilitate the analysis of the statistical properties of EARL, we consider the following alternative estimator based on the sample splitting. Let *I*_1_,*I*_2_, …, *I*_*K*_ denote a random partition of the indices {1,2, …, *n*} with *I*_*j*_ ∩ *I*_*k*_ = ∅ for any *j* ≠ *k* and $\cup_{k=1}^{K}I_{k}=\{1,2,\ldots ,n\}$. We assume the size of the partitions is comparable, that is, *n*_*k*_ = |*I*_*k*_| with *n*_1_ ≍ *n*_2_ ≍ … ≍ *n*_*K*_. In practice, *K* is taken as a small integer (e.g., 2, or 5) and is assumed fixed. Recall that the EARL estimator based on the full sample is defined in (4). In particular, the same samples are used to estimate the nuisance functions $\hat{\pi }$, $\hat{Q}$ and construct the estimator ${\tilde{f}}_{n}^{\lambda_{n}}$ in (4). This creates the delicate dependence between the estimators $\hat{\pi }$, $\hat{Q}$ and the samples used in the empirical risk minimization in (4). To remove this dependence, we now modify the procedure via sample splitting. First, for 1 ≤ *k* ≤ *K*, we construct estimators ${\hat{\pi }}_{k}$, ${\hat{Q}}_{k}$ based on the samples in *I*_*k*_, i.e., {(***X***_*i*_, *A*_*i*_, *Y*_*i*_); *i* ∈ *I*_*k*_}. Denote *I*_(−*k*)_ = {1, …, *n*}\*I*_*k*_. Then, we use the remaining samples *I*_(−*k*)_ for the EARL estimator

(5) $${\hat{f}}_{n,k}^{\lambda_{nk}}=\arg\;\underset{f\in F}{\inf}\;{\mathbb{P}}_{n}^{\left(-k\right)}\left[\left|{\hat{W}}_{1k}\right|\phi \left\{\mathrm{sgn}\left({\hat{W}}_{1k}\right)f(X)\right\}+\left|{\hat{W}}_{-1k}\right|\phi \left\{-\mathrm{sgn}\left({\hat{W}}_{-1k}\right)f(X)\right\}\right]+\lambda_{nk}\|f{\|}^{2},$$

where ${\hat{W}}_{ak}=W_{a}\left(Y,X,A,{\hat{\pi }}_{k},{\hat{Q}}_{k}\right)$, *a* =∈ {−1, 1} and ${{\mathbb{P}}_{n}}^{\left(-k\right)}f=\frac{1}{\left|I_{\left(-k\right)}\right|}\sum_{i\in I_{\left(-k\right)}}f\left(X_{i}\right)$. We note that independent samples are used for estimating the nuisance functions *π, Q* and the decision rule *f*. Thus, the dependence between the estimators $\hat{\pi }$, $\hat{Q}$ and the samples use in (4) is removed. Finally, to obtain a more stable estimator, we can aggregate the estimators

(6) $${\hat{f}}_{n}^{\lambda_{n}}=\frac{1}{K}\sum_{k=1}^{K}{\hat{f}}_{n,k}^{\lambda_{nk}},$$

which is the final estimator based on sample splitting. While the estimator ${\hat{f}}_{n}^{\lambda_{n}}$ requires more computational cost, it has important advantages over the original EARL estimator ${\tilde{f}}_{n}^{\lambda_{n}}$ in (4). From a theoretical perspective, one can still analyze the EARL estimator ${\tilde{f}}_{n}^{\lambda_{n}}$ based on the empirical process theory. This typically requires the entropy conditions on the function classes of *π* and *Q*. In comparison, we show in the following section that the sample splitting estimator ${\hat{f}}_{n}^{\lambda_{n}}$ does not require this condition. To the best of our knowledge, similar sample splitting technique was first applied by Bickel (1982) in general semiparametric estimation problems; see also Schick (1986). Recently, this approach has received attention in causal inference problems as a means of relaxing technical conditions. We refer to Zheng and van der Laan (2011); Chernozhukov et al. (2016); Robins et al. (2017) for further discussion.

## 3. Theoretical properties

Let f*∈M be such that d*(***x***) = sgn{f*(***x***)}, and $V*≜\sup_{f\in M}V(f)=V\left(f*\right)$. Define the population risk of function *f* as

R(f)=E(YI[A≠sgn{f(X)}]/π(A;X)),

and $R^{*}≜\inf_{f\in M}R(f)$. We define the risk in this way to be consistent with the convention that higher risk is less desirable; however, inspection shows that the risk equals *K* – *V* (*f*) where *K* is a constant that does not depend on *f*. Thus, minimizing risk is equivalent to maximizing value, and V*−V(f)=R(f)−R*. Accordingly, for a convex function *ϕ*, we define the *ϕ*-risk

$$R_{\phi }^{m}(f)=E\left[\left|W_{1}^{m}\right|\phi \left\{\mathrm{sgn}\left(W_{1}^{m}\right)f(X)\right\}+\left|W_{-1}^{m}\right|\phi \left\{-\mathrm{sgn}\left(W_{-1}^{m}\right)f(X)\right\}\right].$$

By construction, $R_{\phi }^{m}(f)$ is convex; we assume that it has a unique minimizer and that $R_{\phi }^{m*}≜\inf_{f\in M}R_{\phi }^{m}(f)$. The following result is proved in Web Appendix C.

### Proposition 3.1

*Assume that either π*^*m*^(*a*; ***x***) = *π*(*a*; ***x***) *or Q*^*m*^(***x***, *a*) = *Q*(***x***, *a*). *Define* $\tilde{f}=argmin_{f\in M}R_{\phi }^{m}(f)$ *and c*_*m*_(***x***) = *E*{|*W*_1_(*Y,* ***x***, *A, π*^*m*^, *Q*^*m*^)| + |*W*−_1_(*Y,* ***x***, *A, π*^*m*^, *Q*^*m*^)|}. *Then:*

- $d*(x)=\mathrm{sgn}\{\tilde{f}(x)\};$
- *and*
  $$\psi \left\{\frac{V^{*}-V(f)}{\sup_{x\in {\mathbb{R}}^{p}}c_{m}(x)}\right\}\leq \frac{R_{\phi }^{m}(f)-R_{\phi }^{m*}}{\inf_{x\in {\mathbb{R}}^{p}}c_{m}(x)},$$
  *where ψ*(*θ*) = |*θ*| *for hinge loss,* $\psi (\theta )=1-\sqrt{1-\theta^{2}}$ *for exponential loss, ψ*(*θ*) = (1 + *θ*)log(1 + *θ*)*/*2 + (1 − *θ*)log(1 − *θ*)*/*2 *for logistic loss, and ψ*(*θ*) = *θ*^2^ *for squared hinge loss.*

Part (a) of the preceding proposition states that if either the model for the propensity score or for the *Q*-function is correctly specified, then the EARL procedure, optimized over the space of measurable functions, is Fisher consistent for the optimal rule. Part (b) bounds the difference between *V* (*f*) and *V* * through the surrogate risk difference $R_{\phi }^{m}(f)-R_{\phi }^{m*}$. The different forms of *ψ*(·) are due to the fact that different loss functions induce different distance measures of closeness of *f*(*x*) to the true f*(*x*). We use these risk bounds to derive bounds on the convergence rates of the value of EARL estimators constructd using sample splitting.

Let Π denote the function spaces to which the postulated models for *π*(*a*; ***x***) belong; that is, the estimator $\hat{\pi }(a;x)$ belongs to Π. Similarly, let Q denote a postulated class of models for *Q*(***x***, *a*). In this section, we allow the approximation space, F, to be arbitrary subject to complexity constraints; our results allow both parametric or non-parametric classes of models. Our primary result is a bound on the rate of convergence of $V*-V\left({\hat{f}}_{n}^{\lambda_{n}}\right)$ in terms of the *ϕ*-risk difference $R_{\phi }^{m}\left({\hat{f}}_{n}^{\lambda_{n}}\right)-R_{\phi }^{m*}$.

For any ϵ > 0 and measure *P*, let $N\left\{\epsilon ,F,L_{2}(P)\right\}$ denote the covering number of the space F, that is, $N\left\{\epsilon ,F,L_{2}(P)\right\}$ is the minimal number of closed *L*_2_(*P*)-balls of radius ϵ required to cover F (Kosorok, 2008). Denote $\left\|f{\|}_{P,2}^{2}=Ef^{2}(X\right)$. We make the following assumptions.

### Assumption 2

*There exists M*_*Q*_ > 0 *such that* $|Y|\leq M_{Q}$ *and* $|Q(x,a)|\leq M_{Q}$ *for all* (***x***, *a*) ∈ ℝ^*p*^ × {−1, 1} *and* Q∈Q; *there exists* 0 < *L*_Π_ < *M*_Π_ < 1 *such that L*_Π_ ≤ *π*(*a*; ***x***) ≤ *M*_Π_ *for all* (***x***, *a*) ∈ ℝ^*p*^ × {−1, 1} *and π* ∈ Π.

### Assumption 3

*There exist constants* 0 < *v* < 2 *and c* < ∞ *such that for all* 0 < ϵ ≤ 1: $\sup_{P}\log N\left\{\epsilon ,F,L_{2}(P)\right\}\leq c\epsilon^{-v}$, *where the supremum is taken over all finitely discrete probability measures P.*

### Assumption 4

*For some α, β* > 0, $E{\left\|{\hat{\pi }}_{k}(a;x)-\pi (a;x)\right\|}_{P,2}^{2}=O\left(n^{-2\alpha }\right)$ *and* $E{\left\|{\hat{Q}}_{k}(x,a)-Q(x,a)\right\|}_{P,2}^{2}=O\left(n^{-2\beta }\right)$ *for a* = ±1 *and* 1 ≤ *k* ≤ *K.*

Assumption 2 assumes outcomes are bounded, which often holds in practice. Otherwise, we can always use a large constant to bound the outcome. We also assume propensity scores are bounded away from 0 and 1, which is a standard condition for the identification of the treatment effect in causal inference. Assumption 3 controls the complexity of the function spaces for estimating an optimal ITR. For example, if F is composed of linear combinations of elements in a fixed base class, H, where H has finite Vapnik-Chervonenkis (VC) dimension *vc*, then there exists a constant *c*_*vc*_, depending on *vc*, so that $\sup_{P}\log N\left\{\epsilon ,F,L_{2}(P)\right\}\leq c_{vc}\epsilon^{-2vc/(vc+2)}$ (Theorem 9.4, Kosorok (2008)). We note that the entropy conditions on Q and Π are not needed by using the sample splitting technique, due to the independence between estimating *π, Q* and estimating *f*.

Assumption 4 specifies the rate of convergence of the estimators $\hat{\pi }$ and $\hat{Q}$ in terms of the ‖ · ‖_*P,*2_ norm. It is well known that the *L*_2_ rate of convergence is related to the smoothness of the function classes Q and Π and the dimension of ***X***. For instance, if Q corresponds to the Holder class with smoothness parameter *s* on the domain [0, 1]^*p*^, then Theorem 7 of Newey (1997) implies $E\|\hat{Q}(x,a)-Q(x,a){\|}_{P,2}^{2}=O_{p}\left(K/n+K^{-2s/p}\right)$, where $\hat{Q}(x,a)$ is the regression spline estimator and *K* is the number of basis functions.

Define the approximation error incurred by optimizing over F as

(7) $$A\left(\lambda_{n}\right)=\underset{f\in F}{\inf}\left(\lambda_{n}\|f{\|}^{2}+\sum_{a=\pm 1}E\left[W_{a}^{m}\phi \{a\cdot f(X)\}\right]\right)-\underset{f\in M}{\inf}\sum_{a=\pm 1}E\left[W_{a}^{m}\phi \{a\cdot f(X)\}\right].$$

The following result on the risk bound is the main result in this section and is proved in the Web Appendix D.

### Theorem 3.1

*Suppose that assumptions 1–4 hold, λ*_*n*_ → 0. *Define* $c_{m}(x)=E\{|W_{1}^{m}\|X=x,A=1\}+E\left\{|W_{-1}^{m}\|X=x,A=-1\right\}$. *If Q*^*m*^(***x***, *a*) = *Q*(***x***, *a*) *and π*^*m*^(*a*; ***x***) = *π*(*a*;*x*), *then*

$$\psi \left\{\frac{V^{*}-V\left({\hat{f}}_{n}^{\lambda_{n}}\right)}{\sup_{x\in {\mathbb{R}}^{p}}c_{m}(x)}\right\}≲\frac{1}{\inf_{x\in {\mathbb{R}}^{p}}c_{m}(x)}\cdot [A\left(\lambda_{n}\right)+n^{-\frac{2}{v+2}}\lambda_{n}^{-\frac{v}{v+2}}+n^{-1}\lambda_{n}^{-1}\;\;+\lambda_{n}^{-1/2}n^{-(\alpha +\beta )}+\lambda_{n}^{-1/2}\left(n^{-(1/2+\alpha )}+n^{-(1/2+\beta )}\right)].$$

In all cases considered, the function *ψ* is invertible on [0,1], and its inverse is monotone non-decreasing. Thus, for sufficiently large *n* (making the right-hand-side of the equation sufficiently small) the inequality can be re-arranged to yield a bound on $V*-V\left({\hat{f}}_{n}^{\lambda_{n}}\right)$. The form of *ψ*^−1^ dictates the tightness of the bound as a function of the *ϕ*-risk. According to Lemma 3 in Bartlett et al (2006), a flatter loss function leads to better bound on *ψ* function. In other words, a flatter loss function gives better bounds on *V* * −*V* (*f*) in terms of $R_{\phi }^{m}(f)-R_{\phi }^{m*}$. In this respect, hinge-loss can be seen to provide the tightest bound; however, the *ϕ*-risk is not directly comparable across different loss functions as they are not on the same scale.

The right hand side of the bound in Theorem 3.1 consists of three parts: the approximation error $A\left(\lambda_{n}\right)$ due to the size of the approximation space F, the error $n^{-\frac{2}{v+2}}\lambda_{n}^{-\frac{v}{v+2}}+n^{-1}\lambda_{n}^{-1}$ due to the estimation in the function space F, and the error $\lambda_{n}^{-1/2}n^{-(\alpha +\beta )}+\lambda_{n}^{-1/2}\left(n^{-(1/2+\alpha )}+n^{-(1/2+\beta )}\right)$ incurred from plugging the estimators ${\hat{\pi }}_{k}$ and ${\hat{Q}}_{k}$. As expected, the approximation error decreases as the complexity of the class F increases, whereas the estimation error increases with the complexity of the class F and decreases as the sample size increases.

For the error incurred from plugging the estimators ${\hat{\pi }}_{k}$ and ${\hat{Q}}_{k}$, the component $\lambda_{n}^{-1/2}(n^{-(1/2+\alpha )}+n^{-(1/2+\beta )})$ converges to 0 faster than $\lambda_{n}^{-1/2}n^{-(\alpha +\beta )}$ in regular statistical models (i.e., *α, β* ≤ 1*/*2). Thus, it suffices to only look at the term $\lambda_{n}^{-1/2}n^{-(\alpha +\beta )}$. This term can shrink to 0 sufficiently fast as long as one of the estimators ${\hat{\pi }}_{k}$ and ${\hat{Q}}_{k}$ has a fast rate, due to the multiplicative form of the estimation error. For example, if *α* = *β* = 1*/*4, the error from plugging the estimators $\hat{\pi }$ and $\hat{Q}$ is $n^{-1/2}\lambda_{n}^{-1/2}$. Hence, the rate of the proposed method is faster compared with the outcome weighted learning method, which is developed based on an IPWE and does not enjoy this multiplicative form of the errors. This phenomenon can be viewed as a nonparametric version of the double robustness property (see Fan et al., 2016; Benkeser et al., 2017, for additional discussion). Compared with the results in Athey and Wager (2017), we allow for the surrogate loss to replace the 0–1 loss in solving for the optimizer. While the orders in the bound of convergence rates are comparable, the differences in the constants in the bounds might be due to the application of the surrogate function.

### Remark 1

*If α* = *β and*

(8) $$n^{2\alpha -1}\lambda_{n}^{-1/2}\to \infty ,\;or\;n^{2\alpha (v+2)-2}\lambda_{n}^{1-v/2}\to \infty ,$$

*then*

(9) $$\psi \left\{\frac{V^{*}-V\left({\hat{f}}_{n}^{\lambda_{n}}\right)}{\sup_{x\in {\mathbb{R}}^{p}}c_{m}(x)}\right\}≲\frac{1}{\inf_{x\in {\mathbb{R}}^{p}}c_{m}(x)}\cdot \left[A\left(\lambda_{n}\right)+n^{-\frac{2}{v+2}}\lambda_{n}^{-\frac{v}{v+2}}+n^{-1}\lambda_{n}^{-1}\right],$$

*where the upper bound is of the same order as that obtained if the conditional mean Q*(***x***, *a*) *and propensity score π*(*a*; ***x***) *are known. We note that the additional constraints on α and v in (8) are necessary to obtain the fast rate of convergence (9). For instance, if the function classes* Q *and* Π *are indexed by finite dimensional parameters, we can obtain α* = *β* = 1*/*2 *under mild conditions. As a result, the first condition in (8) holds and the fast rate of convergence (9) is applied. On the other hand, if* F *is a simple class but* $\|\hat{\pi }-\pi {\|}_{P,2}$ *and* $\|\hat{Q}-Q{\|}_{P,2}$ *converge at slower rates, the rate for* $V^{*}-V({\hat{f}}_{n}^{\lambda_{n}})$ *will be driven by* $\lambda_{n}^{-1/2}n^{-(\alpha +\beta )}$.

To estimate the value of the optimal treatment rule *V* *, one can aggregate the empirical value of the sample splitting estimator ${\hat{f}}_{n,k}^{\lambda_{n,k}}$ in each subsamples *I*_(−*k*)_, that is, $\bar{V}=\frac{1}{K}\sum_{k=1}^{K}{\hat{V}}_{\left(-k\right)}\left({\hat{f}}_{n,k}^{\lambda_{n},k}\right)$, where

$${\hat{V}}_{\left(-k\right)}(f)={\mathbb{P}}_{n}^{\left(-k\right)}\left[\left|{\hat{W}}_{1k}\right|\phi \left\{\mathrm{sgn}\left({\hat{W}}_{1k}\right)f(X)\right\}+\left|{\hat{W}}_{-1k}\right|\phi \left\{-\mathrm{sgn}\left({\hat{W}}_{-1k}\right)f(X)\right\}\right],$$

The following corollary, provides a corresponding bound on the rate for $V*-\bar{V}$. The proof is given in Web Appendix D.

### Corollary 3.1

*Suppose that assumptions 1–4 hold, and λ*_*n*_ → 0. *If Q*^*m*^(*x,a*) = *Q*(***x***, *a*) *and π*^*m*^(*a*; ***x***) = *π*(*a*; ***x***), *then*

$$V^{*}-\bar{V}≲\frac{1}{K}\sum_{k=1}^{K}\left[V^{*}-V\left({\hat{f}}_{n,k}^{\lambda_{n,k}}\right)\right]+n^{-1/2}+\lambda_{n}^{-1/2}n^{-(\alpha +\beta )}+\lambda_{n}^{-1/2}\left(n^{-(1/2+\alpha )}+n^{-(1/2+\beta )}\right),$$

*where*

$$\frac{1}{K}\sum_{k=1}^{K}\left[V^{*}-V\left({\hat{f}}_{n,k}^{\lambda_{n,k}}\right)\right]≲\underset{x\in {\mathbb{R}}^{p}}{\sup}c_{m}(x)\psi^{-1}[\frac{1}{\inf_{x\in {\mathbb{R}}^{p}}c_{m}(x)}\cdot \{A\left(\lambda_{n}\right)+n^{-\frac{2}{v+2}}\lambda_{n}^{-\frac{v}{v+2}}+n^{-1}\lambda_{n}^{-1}\;\;+\lambda_{n}^{-1/2}n^{-(\alpha +\beta )}+\lambda_{n}^{-1/2}\left(n^{-(1/2+\alpha )}+n^{-(1/2+\beta )}\right)\}].$$

### Remark 2

*Athey and Wager (2017) and Kitagawa and Tetenov (2017) investigated the binary-action policy learning problem, and established a risk bound of n*^−1/2^ *for both known propensities (Kitagawa and Tetenov, 2017) and unknown propensities (Athey and Wager, 2017). However, they considered a restricted class of decision rules and subsequent risk bound were established with respect to the optimal rule within this restricted class. Hence, there was not consideration of the approximation error. In contrast, we considered the optimal rule within the space consisting of all measurable functions from* ℝ^*p*^ *(the covariate space) to* {−1, 1} *(the treatment space). We used a smaller space, for example, a reproducing kernel Hilbert space, to approximate the policy space and to avoid overfitting. This led to a tradeoff between approximation and estimation error, and λ*_*n*_ *was a tuning parameter to control this bias-variance tradeoff. Consequently, the achieved convergence rates are different.*

## 4. Simulation experiments

We compare EARL estimators with: *Q*-learning fit using ordinary least squares (QL, Qian and Murphy, 2011); estimating the optimal rule within a restricted class based on an AIPW estimator (AIPWE, Zhang et al., 2012b); and outcome weighted learning (OWL, Zhao et al., 2012). Comparisons are made in terms of the average value of the rule estimated by each method. For *Q*-learning, we fit a linear model for the *Q*-function that includes all two-way interactions between predictors and pairwise interactions between these terms and treatment. In the AIPWE method, an AIPW estimator for the value function is constructed and then the optimal linear rule that maximizes the AIPW estimator is identified via a genetic algorithm. Similar to EARL, both a propensity score model and a regression model need to be fitted in AIPWE. We will use the same set of models in EARL and the AIPWE, which are detailed in below. For OWL, we use a linear decision rule; recall that OWL is a special case of EARL with $\hat{Q}(x,a)\equiv 0$, *ϕ*(*t*) = max(0, *t*), and a known propensity score. All estimation methods under consideration require penalization; we choose the amount of penalization using 10-fold cross-validation of the value. Within the class of EARL estimators, we considered hinge, squared-hinge, logistic, and exponential convex surrogates. An implementation of EARL is available in the R package ‘DynTxRegime;’ this package also includes implementations of AIPWE and OWL and therefore can be used to replicate the simulation studies presented here. We included an example for implementing EARL method using ‘DynTxRegime’ package in Web Appendix H.

We consider generative models of the form: *X* = (*X*_1_, …, *X*_*p*_) ∼_*i.i.d.*_ *N*(0,1) with *p* = 10; treatments are binary, taking the values in {−1, 1} according to the model *p*(*A* = 1|***X***) = exp{ℓ(***X***)}*/*[1 + exp{ℓ(***X***)}], where ℓ(***x***) = *x*_1_ + *x*_2_ + *x*_1_*x*_2_ in Scenario 1, and ℓ(***x***) = 0.5*x*_1_ – 0.5 in Scenario 2; $Y=\sum_{j=1}^{p}X_{j}^{2}+\sum_{j=1}^{p}X_{j}+Ac(X)+\epsilon$, where *ϵ* ~ *N*(0, 1), and *c*(***x***) = *x*_1_+*x*_2_−0.1. Write ***X***^2^ to denote $\left(X_{1}^{2},\ldots ,X_{p}^{2}\right)$. The following modeling choices are considered for the propensity and outcome regression models.

CC. A correctly specified logistic regression model for *π*(*A*;***X***) with predictors *X*_1_, *X*_2_ and *X*_1_*X*_2_ in Scenario 1, and with predictor *X*_1_ in Scenario 2; and a correctly specified linear regression model for *Q*(***X***, *A*) with predictors ***X***, ***X***^2^, *A, X*_1_*A* and *X*_2_*A* in both scenarios.
CI. A correctly specified logistic regression model for *π*(*A*; ***X***) with predictors *X*_1_, *X*_2_ and *X*_1_*X*_2_ in Scenario 1, and with predictor *X*_1_ in Scenario 2; and an incorrectly specified linear model for *Q*(***X***, *A*) with predictors ***X***, *A, X****A*** in both scenarios.
IC. An incorrectly specified logistic regression model for *π*(*A*; ***X***) with predictors ***X*** in Scenario 1, and without any predictors in Scenario 2; and a correctly specified linear model for *Q*(***X***, *A*) with predictors ***X***, ***X***^2^, *A, X*_1_*A* and *X*_2_*A* in both scenarios.
II. An incorrectly specified logistic regression model for *π*(*A*; ***X***) with predictors ***X*** in Scenario 1, and without any predictors in Scenario 2; and an incorrectly specified linear model for *Q*(***X***, *A*) with predictors ***X***, *A,* ***X****A* in both scenarios.

We use the same model specifications to carry out AIPWE, and denote them as CC-A, CI-A, IC-A, and II-A correspondingly. For the OWL method, we use correct and incorrect propensity models to construct the ITRs, and denote them as C. and I. respectively. Similarly, we use Q-learning to construct the ITRs based on correct and incorrect regression models, and term them as .C and .I respectively.

**Figure 1:**
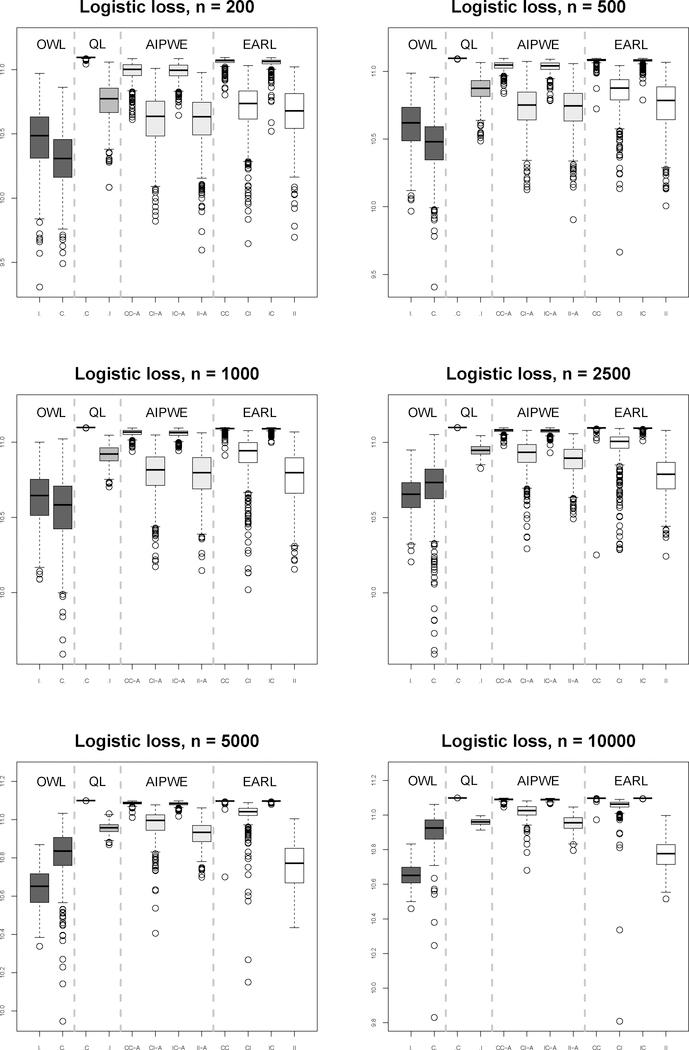
Boxplots for Scenario 1 results under QL, AIPWE, and OWL and EARL using logistic loss.

**Figure 3:**
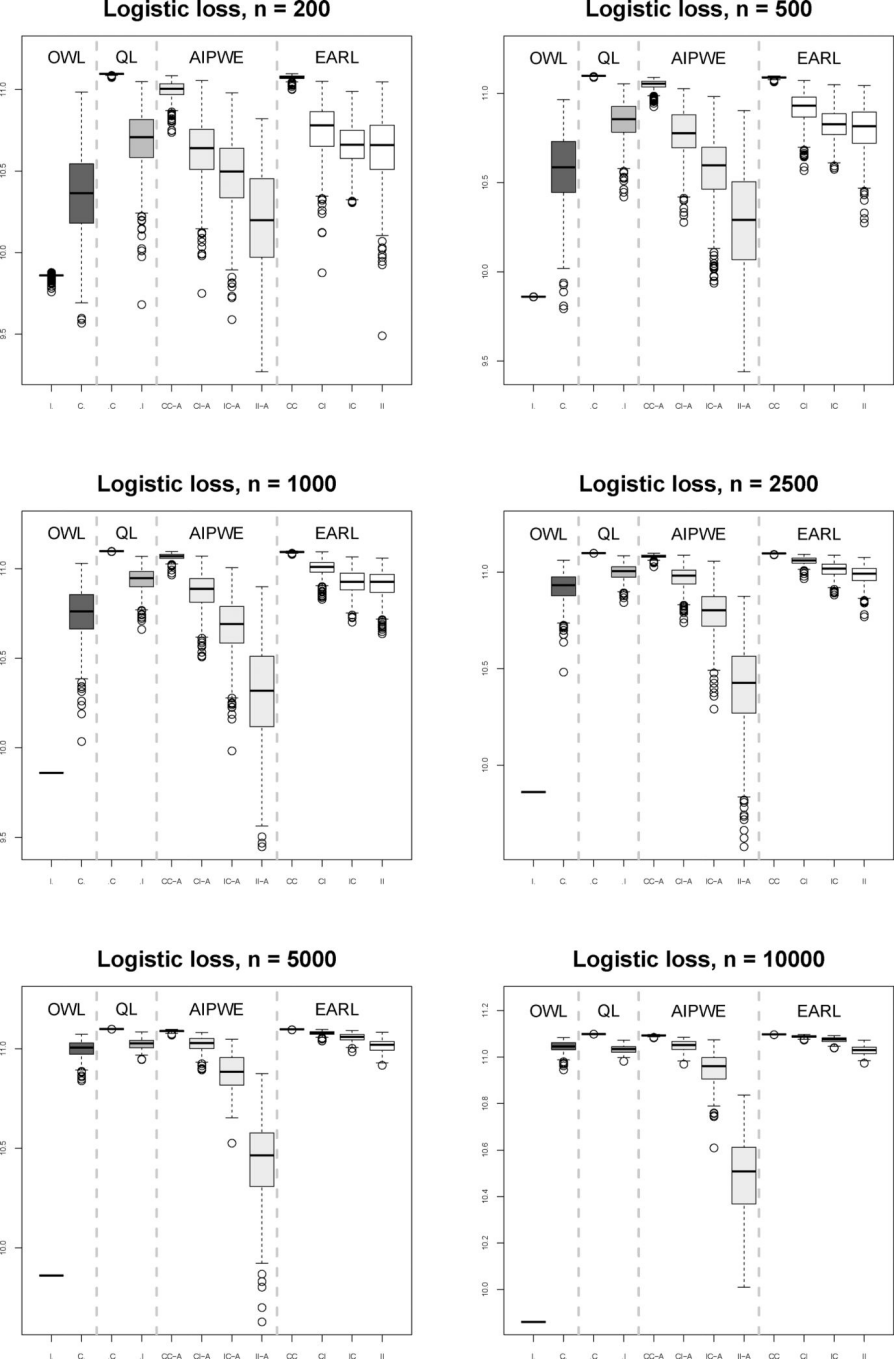
Boxplots for Scenario 3 results under QL, AIPWE, and OWL and EARL using logistic loss.

We consider sample sizes 200, 500, 1000, 2500, 5000 and 10000. We generate a large validation data set (size 10000) and 500 training sets under each sample size. The ITRs are constructed based on one training set out of 500 replicates using competing methods. For implementing EARL, we use logistic loss. We observe similar patterns for other surrogate loss functions (see the Web Appendix). We carry out cross-validation to select *λ*_*n*_ among a pre-specified set of values (2^−5,^,2^−4^, …, 2^5^). Then we calculate the mean response had the whole population followed the rule (the value function) by averaging the outcomes over 10000 subjects under the estimated ITRs in the validation data set. Thus, there are 500 values of the estimated rules on the validation set for each sample size. Boxplots of these values are shown in Figures 1 and 3. The performance of OWL was generally worse than that of the EARL estimator or QL. The AIPWE method exhibits a larger bias and a higher variance compared to the proposed method, while running approximately 200 times slower. As expected, the QL method works best when the model is correctly specified but can perform poorly when this model is misspecified.

It appears that misspecification of the model for the *Q*-function has a bigger impact than misspecification of the propensity score model on the AIPWE and EARL methods. The relatively poor performance when the propensity is correctly specified but the regression model is not might be attributed in part to inverse weighting by the propensity score, which is problematic when some estimated propensity scores are close to zero, yielding large weights and subsequently induces bias (Kang and Schafer, 2007). This is illustrated by contrasting scenarios 1 and 2. Propensity scores in Scenario 2 are bounded away from zero, which yield a better result compared to Scenario 1. Furthermore, the large variability when the regression model is misspecified may be partly a consequence of the method used to estimate the coefficients in the regression model (see Cao et al., 2009).

Finally, we consider an example to illustrate the impact of a severely misspecified propensity score model. In Scenario 3, the data was generated as in Scenario 2 except that the propensity score was set to 0.025 for all subjects. The ‘CI’ setup outperformed the ‘IC’ setup, especially when the sample size was small. Furthermore, the performance of the AIPWE method was largely affected by this poorly imposed propensity model. The results of ‘CI’ and ‘II’ setups were unsatisfactory even when the sample size was increased to 10000. This example indicates that the performances in the ‘CI’ and ‘IC’ setups depend on the degree of misspecification in the outcome regression model and propensity score model.

We also conducted a set of simulation experiments to investigate the role of parametric and nonparametric models for the propensity score and outcome regression. In addition, we compared the performance across different surrogate loss functions, including logistic loss, exponential loss, squared hinge loss, and hinge loss. These additional simulation results can be found in Web Appendix F. In summary, we found that in the examples considered, using nonparametric working models for propensity scores could improve results over parametric models. Hinge loss has a more robust performance when the regression model is incorrect compared to other smooth losses.

## 5. Application: Ocean State Crohn’s and Colitis Area Registry (OSCCAR)

OSCCAR is a community—based incident cohort of subjects with inflammatory bowel disease (IBD) residing in the state of Rhode Island that was established in 2008 (Sands et al., 2009). Subjects enrolled in OSCCAR have ulcerative colitis (UC), Crohn’s disease (CD), or indeterminate colitis (IC). Corticosteroids are commonly used to treat active symptoms. Although, corticosteroids often promptly achieve remission, long-term use is complicated by many potential side effects. One treatment strategy for IBD patients is a “step-up” approach in which patients are prescribed medications with increasing potential toxicity based on the severity of their disease. Alternatively, a “top-down” approach uses aggressive therapy early in the disease course to prevent long-term complications. Both approaches have been shown to be clinically effective, however, there is treatment response heterogeneity and it is not clear which treatment is right for each individual patient. Clinical theory dictates that those likely to experience a more aggressive disease progression would benefit more from “top-down” than “step-up”; whereas those likely to experience a less aggressive progression might benefit more from “step-up.”

The primary outcome is the disease activity score measured at the end of the second year, as measured by the Harvey—Bradshaw Index for subjects with CD and the Simple Clinical Colitis Index for subjects with UC. In both measures, higher scores reflect more disease activity. A high-quality treatment rule would reduce disease activity by assigning patients to top-down if it is necessary and step-up otherwise. Among the 274 patients included in the observed data, 32 patients were assigned to the top-down strategy (*A* = 1) and 242 were assigned to step-up (*A* = −1). To remain consistent with our paradigm of maximizing mean response we used the negative disease activity score as the response, *Y*. 11 patient covariates were used, which included age, gender, ethnicity, marital status, race, body mass index, disease type, antibiotics drug usage, antidiarrheal drug usage, indicator for extra-intestinal manifestation and baseline disease activity scores. We used a linear regression model to estimate the *Q*-function, and a regularized logistic regression model to estimate the propensity score to avoid overfitting. In addition to the EARL estimators we applied QL and OWL to estimate an optimal treatment rule. Because this is an observational study with unknown propensity scores, we evaluated the estimated treatment rules $\hat{d}$ using inverse probability weighting ${\hat{V}}^{\text{IPWE}}(\hat{d})={\mathbb{P}}_{n}[YI\{A=\hat{d}(X)\}/\hat{\pi }(A;X)]/{\mathbb{P}}_{n}[I\{A=\hat{d}(X)\}/\hat{\pi }(A;X)]$, where $\hat{\pi }$ is the estimated propensity score. Higher values of ${\hat{V}}^{\text{IPWE}}(\hat{d})$, that is, lower disease activity scores, indicate a better overall benefit.

The coefficients of the estimated optimal treatment rules constructed from EARL with logistic loss are presented in Table 1. A permutation test based on 2000 permutation times was conducted to obtain the p-value for each covariate, which showed that body mass index was significant at 0.05 level and gender was significant at 0.1 level. In general, patients with a more severe disease status at baseline are likely to benefit from a top-down therapy. This is consistent with clinical theory as these symptoms are associated with higher disease severity.

Table 2 describes the agreement between the estimated optimal decision rules constructed using different methods, which shows that the rules estimated using EARL with different loss functions give quite similar treatment recommendations. In this table, we also present the agreement between the estimated decision rules and the observed treatments. Compared to the observed treatment allocations, the estimated rules encourage more patients to receive top-down therapy, where 161 patients are recommended to top-down treatment by EARL methods with all loss functions, 225 patients are recommended by OWL method using logistic loss and 145 patients are recommended by QL method respectively. The estimated disease activity score is 1.75 using logistic loss, compared with 1.80 for the QL estimator, and 1.75 for OWL using logistic loss. Although the achieved benefit of the ITR yielded by OWL and EARL were similar, EARL recommended less patients to the more intensive top-down therapy, which could benefit patients by reducing the side effects. The achieved benefits of the derived ITRs were greater than the benefit that was achieved in the observed dataset, where the average disease activity score was 2.24. Since top-down therapy is relative new in the practice, to be conservative, physicians tend not to provide such therapy to patients. Our analysis encourages the usage of top-down therapy for a greater benefit, which can be tailored according to individual characteristics. By looking into the relationship between the observed treatment and covariates, we found that in current practice, physicians were more likely to follow top-down therapy while giving out antibiotics and antidiarrheals drugs in patients with Crohn’s disease. The ITRs resulted from EARL, on the other hand, were more likely to recommend top-down therapy for ulcerative colitis/indeterminate colitis patients while they are not taking antibiotics and antidiarrheals drugs.

We also applied our method to the study of National Supported Work Demonstration, which also showed a superior performance of the proposed method. Results are shown in Web Appendix G.

## 6. Discussion

We proposed a class of estimators for the optimal treatment rule that we termed EARL. This class of methods is formed by applying a convex relaxation to the AIPWE of the marginal mean outcome. To reduce the risk of misspecification, it is possible to use flexible, e.g., nonparametric, models for the propensity score and the *Q*-function. However, we showed theoretically and empirically that such flexibility comes at the cost of additional variability and potentially poor small sample performance.

We demonstrated that extreme propensity scores may lead to a large variance in the augmented inverse probability weighted estimator. To alleviate this issue, weight stabilization could potentially help. In particular, we can consider a stabilized weight of the form

$$SW_{a}^{m}=W_{a}^{m}/\frac{I(A=a)}{\pi^{m}(a;X)}.$$

Using modified weight $SW_{a}^{m}$ leads to consistent estimator of the optimal decision rule if positivity assumption holds, that is, Lemma 2.2 still holds under the modified weight. Alternatively, we may also consider an estimator which achieves the smallest variance among its class of doubly robust estimators when the propensity score model is correctly specified. Such an estimator can be derived following the techniques used in Cao et al. (2009).

There are several important ways this work might be extended. The first is to handle time-to-event outcomes wherein the observed data are subject to right-censoring. In this setting, efficient methods for augmentation to adjust for censoring might be folded into the EARL framework. Another extension is to multi-stage treatment rules, also known as, dynamic treatment regimes (Murphy, 2003; Robins, 2004; Moodie et al., 2007). A challenging component of this extension is that the variability of the AIPWE increases dramatically as the number of treatment stages increases. We believe that the convex relaxation may help in this setting not only in terms of computation but also by reducing variance.

## Supplementary Material

Supplementary material

## Figures and Tables

**Figure 2:**
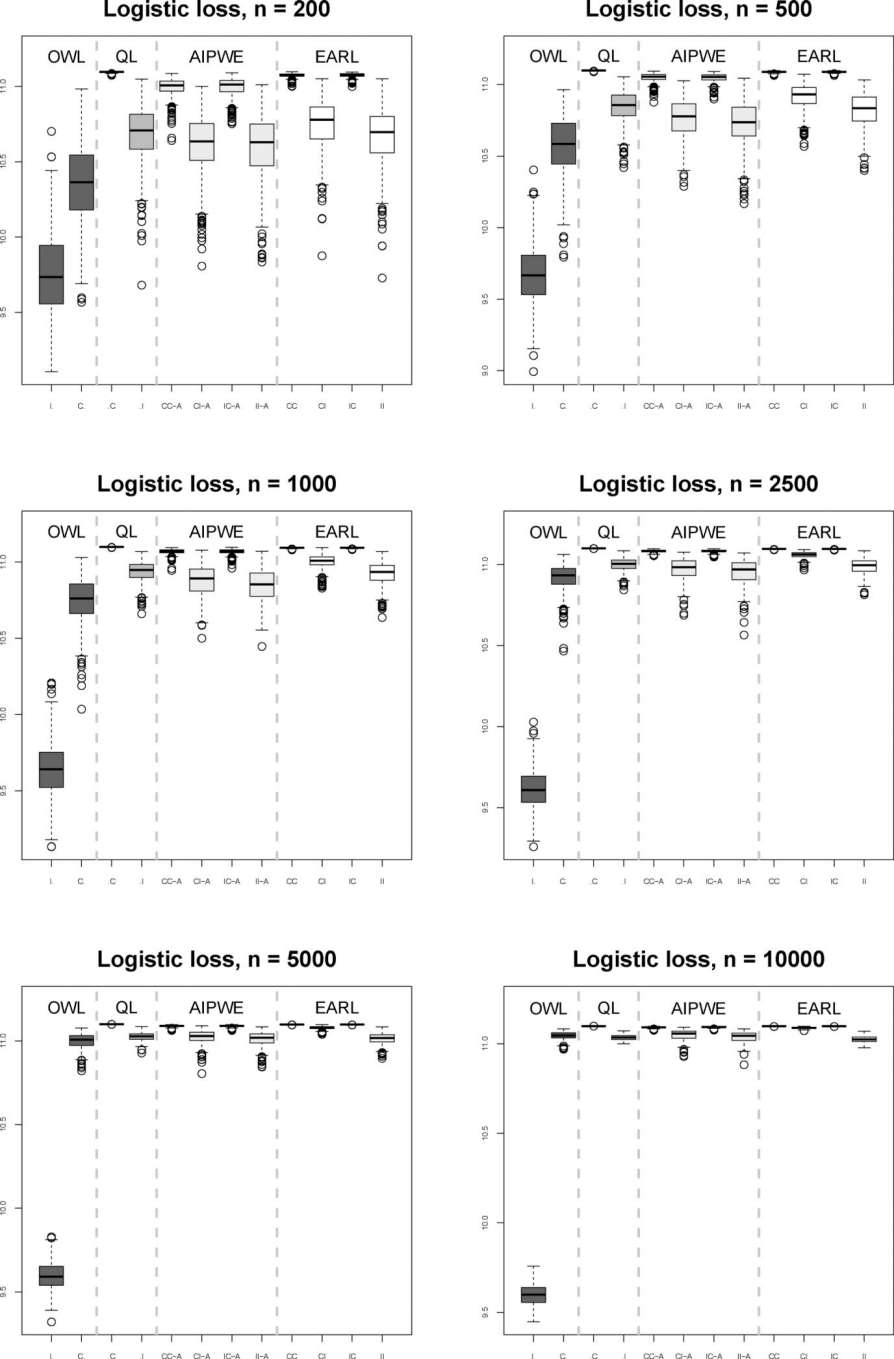
Boxplots for Scenario 2 results under QL, AIPWE, and OWL and EARL using logistic loss.

**Table 1:** Coefficients for the estimated optimal decision rules by EARL with logistic loss (*: significant at 0.05 level).

	Coefficient	p-value
Intercept	2.466	-
Age	−0.001	0.905
Gender (Male = 1)	0.756	0.015*
Ethnicity (Hispanic = 1)	−1.045	0.144
Marital status (Single = 1)	−0.320	0.318
Race (White = 1)	−0.233	0.478
Body mass index	−0.063	0.037*
Disease type (UC or IC = 1)	0.309	0.234
Antibiotics drug usage (Yes = 1)	−0.156	0.563
Antidiarrheals drug usage (Yes = 1)	−0.580	0.167
Extra-intestinal manifestation (Yes = 1)	0.273	0.286
Baseline disease activity scores	0.050	0.427

**Table 2:** Agreements between the estimated optimal decision rule yield by different methods and the observed treatment. OWL-logit: OWL using logistic loss; EARL: EARL using logistic loss; EARL: EARL using exponential oss; EARL-hinge: EARL using hinge loss; EARL-sqhinge: EARL using squared hinge loss; QL: Q-learning.

	OWL-Logit	EARL-logit	EARL-exp	EARL-hinge	EARL-sqhinge	QL
OWL-Logit	1	0.642	0.821	0.639	0.639	0.577
EARL-logit		1	0.588	0.996	0.996	0.920
EARL-exp			1	0.591	0.591	0.529
EARL-hinge				1	1	0.916
EARL-sqhinge					1	0.916
QL						1
Observed	0.193	0.449	0.117	0.453	0.453	0.507
