# Supplementary material for: Efficient augmentation and relaxation learning for individualized treatment rules using observational data
Source: J Mach Learn Res. Author manuscript; Available in PMC 2019 Aug 22. (PMC6705615)

Web-based Supplementary Materials for "Efficient augmentation and relaxation learning for individualized treatment rules using observational data"

Ying-Qi Zhao YQZHAO@FREDHUTCH.ORG
Public Health Sciences Division
Fred Hutchinson Cancer Research Center
Seattle, WA, 98109, USA

Eric B. Laber EBLABER@NCSU.EDU
Department of Statistics
North Carolina State University
Raleigh, NC, 27695, USA

Yang Ning YN265@CORNELL.EDU
Department of Statistical Science
Cornell University
Ithaca, NY, 14853, USA

Sumona Saha SSAHA@MEDICINE.WISC.EDU
School of Medicine and Public Health
University of Wisconsin
Madison, WI, 53705, USA

Bruce E. Sands BRUCE.SANDS@MSSM.EDU
Division of Gastroenterology
Icahn School of Medicine at Mount Sinai
New York, NY, 10029, USA

Editor: Francis Bach and David Blei and Bernhard Schlkopf

Web Appendix A: Proof of Lemma 2.1

Recall that $Q(\boldsymbol{x}, a) = E(Y|A = a, \boldsymbol{X} = \boldsymbol{x})$ and $\pi(a; \boldsymbol{x}) = P(A = a|\boldsymbol{X} = \boldsymbol{x})$. Let $\pi^m(a; \boldsymbol{x})$ and $Q^m(\boldsymbol{x}, a)$ be the limit of $\widehat{\pi}(a; \boldsymbol{x})$ and $\widehat{Q}(\boldsymbol{x}, a)$ respectively. If $\widehat{\pi}(a; \boldsymbol{x}) \to_p \pi(a; \boldsymbol{x})$, i.e., $\pi^m(a; \boldsymbol{x}) = \pi(a; \boldsymbol{x})$, then $\widehat{V}^{AIPWE}(d)$ is equal to

$$\mathbb{P}_n \left[\frac{Y}{\pi(A; \boldsymbol{X})} I\left\{A = d(\boldsymbol{X})\right\} - \frac{I\left\{A = d(\boldsymbol{X})\right\} - \pi\{d(\boldsymbol{X}); \boldsymbol{X}\}}{\pi\{d(\boldsymbol{X}); \boldsymbol{X}\}} Q^m\{\boldsymbol{X}, d(\boldsymbol{X})\} \right] + o_p(1),$$

which converges to

$$\begin{aligned} V^{AIPWE,m}(d) &= V(d) - E\Big[\frac{I\{A = d(\boldsymbol{X})\} - \pi\{d(\boldsymbol{X}); \boldsymbol{X}\}}{\pi\{d(\boldsymbol{X}); \boldsymbol{X}\}} Q^m\{\boldsymbol{X}, d(X)\}\Big] \\ &= V(d) - E\Big(Q^m\{\boldsymbol{X}, d(X)\} E\Big[\frac{I\{A = d(\boldsymbol{X})\} - \pi\{d(\boldsymbol{X}); \boldsymbol{X}\}}{\pi\{d(\boldsymbol{X}); \boldsymbol{X}\}} |\boldsymbol{X}, d(\boldsymbol{X})\Big]\Big) = V(d). \end{aligned}$$

Here we have used the fact that $E[I\{A = d(\boldsymbol{X})\}|\boldsymbol{X}, d(\boldsymbol{X})] = P\{A = d(\boldsymbol{X})|\boldsymbol{X}\} = \pi\{d(\boldsymbol{X}); \boldsymbol{X}\}$.

If $\widehat{Q}(\boldsymbol{x}, a) \to_p Q(\boldsymbol{x}, a)$, i.e., $Q^m(\boldsymbol{x}, a) = Q(\boldsymbol{x}, a)$, $\widehat{V}^{AIPWE}(d)$ is equal to

$$\mathbb{P}_n\left[\frac{Y}{\pi^m\{d(\boldsymbol{X}); \boldsymbol{X}\}} I\left\{A = d(\boldsymbol{X})\right\} - \frac{I\left\{A = d(\boldsymbol{X})\right\} - \pi^m\{d(\boldsymbol{X}); \boldsymbol{X}\}}{\pi^m\{d(\boldsymbol{X}); \boldsymbol{X}\}} Q(\boldsymbol{X}, d(\boldsymbol{X}))\right] + o_p(1),$$

which converges to

$$\begin{aligned} V^{AIPWE,m}(d) &= E\Big[\frac{I\{A = d(\boldsymbol{X})\}}{\pi^m(A; \boldsymbol{X})} Y - \frac{I\{A = d(\boldsymbol{X})\} - \pi^m\{d(\boldsymbol{X}); \boldsymbol{X}\}}{\pi^m\{d(\boldsymbol{X}); \boldsymbol{X}\}} E\{Y|\boldsymbol{X}, A = d(\boldsymbol{X})\}\Big] \\ &= E\Big[\frac{E[I\{A = d(\boldsymbol{X})\}Y|\boldsymbol{X}, A] - E[I\{A = d(\boldsymbol{X})\}E\{Y|\boldsymbol{X}, A = d(\boldsymbol{X})\}|\boldsymbol{X}, A]}{\pi^m\{d(\boldsymbol{X}); \boldsymbol{X}\}}\Big] \\ &+ E[E\{Y|\boldsymbol{X}, A = d(\boldsymbol{X})\}] \end{aligned}$$

Note that the first term in the above equation is equal to zero, and according to (1), the second term is equal to $V(d)$. Hence, we have obtained the desired result. $\square$

Web Appendix B: Proof of Lemma 2.2

As $V^{AIPWE,m}(d) = E[W_1^m I\{d(\boldsymbol{X}) = 1\} + W_{-1}^m I\{d(\boldsymbol{X}) = -1\}]$, it follows that $\widehat{V}^{AIPWE}(f) = \mathbb{P}_n \widehat{W}_1 I\left\{f(\boldsymbol{X}) \geq 0\right\} + \mathbb{P}_n \widehat{W}_{-1} I\left\{-f(\boldsymbol{X}) \geq 0\right\}$, and

$$\widehat{f}_n = \arg\inf_{f \in \mathcal{F}} [\mathbb{P}_n \widehat{W}_1 I\left\{f(\boldsymbol{X}) < 0\right\} + \mathbb{P}_n \widehat{W}_{-1} I\left\{-f(\boldsymbol{X}) < 0\right\}].$$

If $f(\boldsymbol{x}) = 0$, then $\mathbb{P}_n \widehat{W}_1 I\left\{f(\boldsymbol{X}) < 0\right\} + \mathbb{P}_n \widehat{W}_{-1} I\left\{-f(\boldsymbol{X}) < 0\right\} = \mathbb{P}_n |\widehat{W}_1| I\{\text{sgn}(\widehat{W}_1) f(\boldsymbol{X}) < 0\} + \mathbb{P}_n |\widehat{W}_{-1}| I\{-\text{sgn}(\widehat{W}_{-1}) f(\boldsymbol{X}) < 0\}$. Otherwise, define $\text{sgn}(0) = 1$, and for any constants w and $f \neq 0$,

$$\begin{aligned} |w| I\{\text{sgn}(w) f < 0\} &= w I\{\text{sgn}(f) \neq 1\} I(w \geq 0) - w I\{\text{sgn}(f) = 1\} I(w < 0) \\ &= w I\{\text{sgn}(f) \neq 1\} I(w \geq 0) - w[1 - I\{\text{sgn}(f) \neq 1\}] I(w < 0) \\ &= w I(f < 0) - w I(w < 0). \end{aligned}$$

Therefore, for $a = \pm 1$,

$$\mathbb{P}_n |\widehat{W}_a| I\{a \cdot \text{sgn}(\widehat{W}_a) f(\boldsymbol{X}) < 0\} = \mathbb{P}_n \widehat{W}_a I\{a \cdot f(\boldsymbol{X}) < 0\} - \mathbb{P}_n \widehat{W}_a I(\widehat{W}_a < 0).$$

The second term in the right hand side is a constant that does not affect the estimation of the rule.

Web Appendix C: Proof of Proposition 3.1

Recall that $Q(\boldsymbol{x}, a) = E(Y|\boldsymbol{X} = \boldsymbol{x}, A = a)$ and $\pi(a; \boldsymbol{x}) = P(A = a|\boldsymbol{X} = \boldsymbol{x})$. Define

$$W_a^* = W_a(Y, \boldsymbol{X}, A, \pi, Q) = I(A = a)\frac{Y - Q(\boldsymbol{X}, a)}{\pi(a; \boldsymbol{X})} + Q(\boldsymbol{X}, a), a = \pm 1.$$

(a) We first consider hinge loss, $\phi(t) = \max(1-t, 0)$. Note that,

$$
\begin{aligned}
& |W_1^*|\phi\left(\text{sgn}(W_1^*)f(\boldsymbol{X})\right) + |W_{-1}^*|\phi\left(-\text{sgn}(W_{-1}^*)f(\boldsymbol{X})\right) \\
= \; & W_1^* \max\{1-f(\boldsymbol{X}), 0\} I(W_1^* \geq 0) - W_1^* \max\{1+f(\boldsymbol{X}), 0\} I(W_1^* < 0) \\
& + W_{-1}^* \max\{1+f(\boldsymbol{X}), 0\} I(W_{-1}^* \geq 0) - W_{-1}^* \max\{1-f(\boldsymbol{X}), 0\} I(W_{-1}^* < 0)
\end{aligned}
$$

For each $\boldsymbol{x}$, we seek the minimizer $f(\boldsymbol{x})$ of $E\{|W_1^*|\phi\left(\text{sgn}(W_1^*)f(\boldsymbol{X})\right) + |W_{-1}^*|\phi\left(-\text{sgn}(W_{-1}^*)f(\boldsymbol{X})\right)|\boldsymbol{X} = \boldsymbol{x}\}$. Then, the value $f(\boldsymbol{x})$ should be in $[-1, 1]$ because otherwise truncation of f at -1 or 1 gives a lower loss value. When $-1 \leq f(\boldsymbol{x}) \leq 1$,

$$
\begin{aligned}
& E\{|W_1^*|\phi\left(\text{sgn}(W_1^*)f(\boldsymbol{X})\right) + |W_{-1}^*|\phi\left(-\text{sgn}(W_{-1}^*)f(\boldsymbol{X})\right)|\boldsymbol{X} = \boldsymbol{x}\} \\
= \; & \{E(W_{-1}^*|\boldsymbol{X} = \boldsymbol{x}) - E(W_1^*|\boldsymbol{X} = \boldsymbol{x})\} f(\boldsymbol{x}) \\
& + E(W_1^* I(W_1^* \geq 0)|\boldsymbol{X} = \boldsymbol{x}) - E(W_1^* I(W_1^* < 0)|\boldsymbol{X} = \boldsymbol{x}) \\
& + E(W_{-1}^* I(W_{-1}^* \geq 0)|\boldsymbol{X} = \boldsymbol{x}) - E(W_{-1}^* I(W_{-1}^* < 0)|\boldsymbol{X} = \boldsymbol{x}).
\end{aligned}
$$

Therefore, $\text{sgn}\{\tilde{f}(\boldsymbol{x})\} = \text{sgn}\{E(W_1^*|\boldsymbol{X} = \boldsymbol{x}) - E(W_{-1}^*|\boldsymbol{X} = \boldsymbol{x})\}$. Note that for $a = \pm 1$,

$$
\begin{aligned}
E(W_a^*|\boldsymbol{X} = \boldsymbol{x}) &= E\left\{ I(A = a) \frac{Y - Q(\boldsymbol{X}, a)}{\pi(a; \boldsymbol{X})} \middle| \boldsymbol{X} = \boldsymbol{x} \right\} + Q(\boldsymbol{x}, a) \\
&= E\left\{ I(A = a) \frac{E(Y|A = a, \boldsymbol{X} = \boldsymbol{x}) - Q(\boldsymbol{x}, a)}{\pi(a; \boldsymbol{x})} \middle| \boldsymbol{X} = \boldsymbol{x} \right\} + Q(\boldsymbol{x}, a) \\
&= E(Y|\boldsymbol{X} = \boldsymbol{x}, A = a). \qquad (1)
\end{aligned}
$$

Thus, $\text{sgn}\{\tilde{f}(\boldsymbol{x})\} \equiv d^*(\boldsymbol{x})$.

We now consider logistic loss $\phi(t) = \log(1 + e^{-t})$.

$$
\begin{aligned}
& |W_1^*|\phi\left(\text{sgn}(W_1^*)f(\boldsymbol{X})\right) + |W_{-1}^*|\phi\left(-\text{sgn}(W_{-1}^*)f(\boldsymbol{X})\right) \\
= \; & \log\{1 + e^{-f(\boldsymbol{X})}\} W_1^* I(W_1^* \geq 0) - \log\{1 + e^{f(\boldsymbol{X})}\} W_1^* I(W_1^* < 0) \\
& + \log\{1 + e^{f(\boldsymbol{X})}\} W_{-1}^* I(W_{-1}^* \geq 0) - \log\{1 + e^{-f(\boldsymbol{X})}\} W_{-1}^* I(W_{-1}^* < 0).
\end{aligned}
$$

For each $\boldsymbol{X} = \boldsymbol{x}$,

$$
\begin{aligned}
& E[|W_1^*|\phi\left(\text{sgn}(W_1^*)f(\boldsymbol{X})\right) + |W_{-1}^*|\phi\left(-\text{sgn}(W_{-1}^*)f(\boldsymbol{X})\right)|\boldsymbol{X} = \boldsymbol{x}] \\
= \; & \log\{1 + e^{-f(\boldsymbol{x})}\} E(W_1^* I(W_1^* \geq 0)|\boldsymbol{X} = \boldsymbol{x}) - \log\{1 + e^{f(\boldsymbol{x})}\} E(W_1^* I(W_1^* < 0)|\boldsymbol{X} = \boldsymbol{x}) \\
& + \log\{1 + e^{f(\boldsymbol{x})}\} E(W_{-1}^* I(W_{-1}^* \geq 0)|\boldsymbol{X} = \boldsymbol{x}) - \log\{1 + e^{-f(\boldsymbol{x})}\} E(W_{-1}^* I(W_{-1}^* < 0)|\boldsymbol{X} = \boldsymbol{x}).
\end{aligned}
$$

Take the derivative with respect to f and set it to zero to obtain

$$
\tilde{f}(\boldsymbol{x}) = \log \frac{E(W_1^* I(W_1^* \geq 0)|\boldsymbol{X} = \boldsymbol{x}) - E(W_{-1}^* I(W_{-1}^* < 0)|\boldsymbol{X} = \boldsymbol{x})}{-E(W_1^* I(W_1^* < 0)|\boldsymbol{X} = \boldsymbol{x}) + E(W_{-1}^* I(W_{-1}^* \geq 0)|\boldsymbol{X} = \boldsymbol{x})},
$$

which is positive if $E(W_1^*|\boldsymbol{X} = \boldsymbol{x}) \geq E(W_{-1}^*|\boldsymbol{X} = \boldsymbol{x})$. Thus, it has the same sign as $d^*(\boldsymbol{x})$.

In the case of exponential loss $\phi(t) = e^{-t}$, for each $\boldsymbol{x}$,

$$
\begin{aligned}
& E[|W_1^*|\phi\left(\text{sgn}(W_1^*)f(\boldsymbol{X})\right) + |W_{-1}^*|\phi\left(-\text{sgn}(W_{-1}^*)f(\boldsymbol{X})\right)|\boldsymbol{X} = \boldsymbol{x}] \\
= \; & e^{-f(\boldsymbol{x})} E(W_1^* I(W_1^* \geq 0)|\boldsymbol{X} = \boldsymbol{x}) - e^{f(\boldsymbol{x})} E(W_1^* I(W_1^* < 0)|\boldsymbol{X} = \boldsymbol{x}) \\
+ \; & e^{f(\boldsymbol{x})} E(W_{-1}^* I(W_{-1}^* \geq 0)|\boldsymbol{X} = \boldsymbol{x}) - e^{-f(\boldsymbol{x})} E(W_{-1}^* I(W_{-1}^* < 0)|\boldsymbol{X} = \boldsymbol{x}).
\end{aligned}
$$

Take the derivative with respect to f and set it to zero, to obtain

$$\tilde{f}(\boldsymbol{x}) = \frac{1}{2}\log\frac{E(W_1^* I(W_1^* \geq 0)|\boldsymbol{X}=\boldsymbol{x}) - E(W_{-1}^* I(W_{-1}^* < 0)|\boldsymbol{X}=\boldsymbol{x})}{-E(W_1^* I(W_1^* < 0)|\boldsymbol{X}=\boldsymbol{x}) + E(W_{-1}^* I(W_{-1}^* \geq 0)|\boldsymbol{X}=\boldsymbol{x})},$$

which is positive if $E(W_1^*|\boldsymbol{X}=\boldsymbol{x}) \geq E(W_{-1}^*|\boldsymbol{X}=\boldsymbol{x})$. Thus, it has the same sign as $d^*(\boldsymbol{x})$.

Finally, if $\phi(t) = \{\max(1-t, 0)\}^2$, then for each $\boldsymbol{x}$,

$$\begin{aligned} & E[|W_1^*|\phi\left(\mathrm{sgn}(W_1^*)f(\boldsymbol{X})\right) + |W_{-1}^*|\phi\left(-\mathrm{sgn}(W_{-1}^*)f(\boldsymbol{X})\right)|\boldsymbol{X}=\boldsymbol{x}] \\ = & E(W_1^* I(W_1^* \geq 0)|\boldsymbol{X}=\boldsymbol{x})\{1-f(\boldsymbol{x})\}^2 - E(W_1^* I(W_1^* < 0)|\boldsymbol{X}=\boldsymbol{x})\{1+f(\boldsymbol{x})\}^2 \\ & + E(W_{-1}^* I(W_{-1}^* \geq 0)|\boldsymbol{X}=\boldsymbol{x})\{1+f(\boldsymbol{x})\}^2 \\ & - E(W_{-1}^* I(W_{-1}^* > 0)|\boldsymbol{X}=\boldsymbol{x})\{1-f(\boldsymbol{x})\}^2. \end{aligned}$$

Take the derivative with respect to f and set it to zero to obtain $\tilde{f}(\boldsymbol{x}) = E(W_1^*|\boldsymbol{X}=\boldsymbol{x}) - E(W_{-1}^*|\boldsymbol{X}=\boldsymbol{x})$, the sign of which is the same as $d^*(\boldsymbol{x})$. □

(b) Without loss of generality, we assume that W_1^m and W_{-1}^m are nonnegative. If not, we can always transform Y to $Y + C$ and $Q(\cdot)$ to $Q(\cdot) + C$ for an arbitrary constant $C > 0$ such that W_1^m and W_{-1}^m are nonnegative with probability tending to one. Note that $E(W_a^m|\boldsymbol{X}=\boldsymbol{x}) = E(W_a^*|\boldsymbol{X}=\boldsymbol{x})$ if either $Q^m(\boldsymbol{x}, a) = Q(\boldsymbol{x}, a)$ or $\pi^m(a;\boldsymbol{x}) = \pi(a;\boldsymbol{x})$. Then $c_m(\boldsymbol{x}) = E(W_1^*|\boldsymbol{X}=\boldsymbol{x}) + E(W_{-1}^*||\boldsymbol{X}=\boldsymbol{x})$. Let $\eta(\boldsymbol{x}) = E(W_1^*|\boldsymbol{X}=\boldsymbol{x})/c_m(\boldsymbol{x})$. It follows that $2\eta(\boldsymbol{x}) - 1 = \{E(W_1^*|\boldsymbol{X}=\boldsymbol{x}) - E(W_{-1}^*|\boldsymbol{X}=\boldsymbol{x})\}/c_m(\boldsymbol{x})$. Recall that $\mathcal{R}(f)$ and $\mathcal{R}_\phi^m(f)$ are defined in the beginning of Section 3. Therefore,

$$\mathcal{R}(f) = E[c_m(\boldsymbol{X})(\eta(\boldsymbol{X})I(\mathrm{sgn}(f(\boldsymbol{X})) \neq 1) + (1-\eta(\boldsymbol{X}))I(\mathrm{sgn}(f(\boldsymbol{X})) \neq -1))],$$

and

$$\mathcal{R}_\phi^m(f) = E\left[c_m(\boldsymbol{X})(\eta(\boldsymbol{X})\phi(f(\boldsymbol{X})) + (1-\eta(\boldsymbol{X}))\phi(-f(\boldsymbol{X})))\right].$$

Note that $\mathcal{R}^* = \mathcal{R}\{\eta(\boldsymbol{X}) - 1/2]\}$, so that

$$\begin{aligned} \mathcal{R}(f) - \mathcal{R}^* &= E\left[(I[\mathrm{sgn}\{f(\boldsymbol{X})\} \neq 1] - I[\mathrm{sgn}\{\eta(\boldsymbol{X}) - 1/2\} \neq 1)])c_m(\boldsymbol{X})\{2\eta(\boldsymbol{X}) - 1\}\right] \\ &= E\left(I[\mathrm{sgn}\{f(\boldsymbol{X})\} \neq \mathrm{sgn}\{\eta(\boldsymbol{X}) - 1/2\}]c_m(\boldsymbol{X})\left|2\eta(\boldsymbol{X}) - 1\right|\right). \end{aligned} \tag{2}$$

The second equality follows because $(I[\mathrm{sgn}\{f(\boldsymbol{X})\} \neq 1] - I[\mathrm{sgn}\{\eta(\boldsymbol{X}) - 1/2\} \neq 1)])\{2\eta(\boldsymbol{X}) - 1\} = I[\mathrm{sgn}\{f(\boldsymbol{X})\} \neq \mathrm{sgn}\{\eta(\boldsymbol{X}) - 1/2\}]\{2\eta(\boldsymbol{X}) - 1\}$ if $2\eta(\boldsymbol{X}) - 1 \geq 0$ and $-I[\mathrm{sgn}\{f(\boldsymbol{X})\} \neq \mathrm{sgn}\{\eta(\boldsymbol{X}) - 1/2\}]\{2\eta(\boldsymbol{X}) - 1\}$ if $2\eta(\boldsymbol{X}) - 1 < 0$.

Define $C(\eta, \alpha) = \eta\phi(\alpha) + (1-\eta)\phi(-\alpha)$, then $\mathcal{R}_\phi^{m*} = E\left[c_m(\boldsymbol{X})\inf_{\alpha\in\mathbb{R}} C(\eta(\boldsymbol{X}), \alpha)\right]$ and

$$\mathcal{R}_\phi^m(f) - \mathcal{R}_\phi^{m*} = E\left[c_m(\boldsymbol{X})\left(C(\eta(\boldsymbol{X}), f(\boldsymbol{X})) - \inf_{\alpha\in\mathbb{R}} C(\eta(\boldsymbol{X}), \alpha)\right)\right].$$

By applying the result in Bartlett et al. (2006) for transforms of different surrogate losses, we have

$$\psi(2\eta - 1) = \inf_{\alpha:\alpha(2\eta-1)\leq 0} C(\eta, \alpha) - \inf_{\alpha\in\mathbb{R}} C(\eta, \alpha). \tag{3}$$

The specific forms of ψ for different convex loss functions can be calculated using the method described in Bartlett et al. (2006), and thus details are omitted. According to (2) and (3), we have

$$\begin{aligned}
&\psi\left\{\frac{\mathcal{R}(f)-\mathcal{R}^*}{\sup_{\boldsymbol{x}\in\mathbb{R}^p} c_m(\boldsymbol{x})}\right\} \leq \psi[E\{I(\mathrm{sgn}(f(\boldsymbol{X}))\neq \mathrm{sgn}[\eta(\boldsymbol{X})-1/2])(2\eta(\boldsymbol{X})-1)\}] \\
&\leq E\left(I(\mathrm{sgn}(f(\boldsymbol{X}))\neq \mathrm{sgn}[\eta(\boldsymbol{X})-1/2])\psi(|(2\eta(\boldsymbol{X})-1)|)\right) \\
&= E\Big(I[\mathrm{sgn}(f(\boldsymbol{X}))\neq \mathrm{sgn}\{\eta(\boldsymbol{X})-1/2\}]\Big[\inf_{\alpha:\alpha(2\eta(\boldsymbol{X})-1)\leq 0} C(\eta(\boldsymbol{X}),\alpha)-\inf_{\alpha\in\mathbb{R}} C(\eta(\boldsymbol{X}),\alpha)\Big]\Big) \\
&\leq E\Big[C(\eta(\boldsymbol{X}),f(\boldsymbol{X}))-\inf_{\alpha\in\mathbb{R}} C(\eta(\boldsymbol{X}),\alpha)\Big] \\
&\leq \frac{1}{\inf_{\boldsymbol{x}\in\mathbb{R}^p} c_m(\boldsymbol{x})} E\left[c_m(\boldsymbol{X})\left(C(\eta(\boldsymbol{X}),f(\boldsymbol{X}))-\inf_{\alpha\in\mathbb{R}} C(\eta(\boldsymbol{X}),\alpha)\right)\right] \\
&= \frac{1}{\inf_{\boldsymbol{x}\in\mathbb{R}^p} c_m(\boldsymbol{x})}[\mathcal{R}_\phi^m(f)-\mathcal{R}_\phi^{m*}].\square
\end{aligned}$$

Web Appendix D: Proof of Theorem 3.1

To make the dependence on the models for the propensity score and Q-function explicit, we write $V^{AIPWE,m}(d)$ as $V_R(f,\pi^m,Q^m)$ for a given $d(\boldsymbol{x})=\mathrm{sgn}\{f(\boldsymbol{x})\}$. For notational simplicity, we use $\hat{f}$ to denote $\hat{f}_n^{\lambda_n}$. Note that for any f, $V(f)=V_R(f,\pi,Q)$. Therefore

$$\begin{aligned}
V^*-V(\widehat{f}) &= V(f^*)-\sup_{f\in\mathcal{F}} V_R(f,\pi^m,Q^m)+\sup_{f\in\mathcal{F}} V_R(f,\pi^m,Q^m)-V_R(\widehat{f},\pi^m,Q^m) \\
&\quad + V_R(\widehat{f},\pi^m,Q^m)-V(\widehat{f}) \\
&\leq V(f^*)-V_R(f^*,\pi^m,Q^m)+\{\sup_{f\in\mathcal{F}} V_R(f,\pi^m,Q^m)-V_R(\widehat{f},\pi^m,Q^m)\} \\
&\quad + V_R(\widehat{f},\pi^m,Q^m)-V(\widehat{f}) \\
&\leq \sup_{f\in\mathcal{F}} V_R(f,\pi^m,Q^m)-V_R(\widehat{f},\pi^m,Q^m)+2\sup_{f\in\mathcal{F}}|V_R(f,\pi,Q)-V_R(f,\pi^m,Q^m)|.
\end{aligned}$$

The first inequality follows because $V_R(f^*,\pi^m,Q^m)\leq \sup_{f\in\mathcal{F}} V_R(f,\pi^m,Q^m)$. If either $\pi^m(a;\boldsymbol{x})=\pi(a;\boldsymbol{x})$ or $Q^m(\boldsymbol{x},a)=Q(\boldsymbol{x},a)$, then $\sup_{f\in\mathcal{F}}|V_R(f,\pi,Q)-V_R(f,\pi^m,Q^m)|=0$ (see Lemma 2.1). Thus, $V^*-V(\widehat{f})\leq \sup_{f\in\mathcal{F}} V_R(f,\pi^m,Q^m)-V_R(\widehat{f},\pi^m,Q^m)$.

Without loss of generality, we assume that $W_a^m, a=\pm 1$, are nonnegative. Let $f^m=\mathrm{argmin}_{f\in\mathcal{M}} E[W_1^m\phi\{f(\boldsymbol{X})\}+W_{-1}^m\phi\{-f(\boldsymbol{X})\}]$. Because $V_R(f^m,\pi^m,Q^m)=\sup_{f\in\mathcal{M}} V_R(f,\pi^m,Q^m)$, it suffices to derive the convergence rate of $V_R(f^m,\pi^m,Q^m)-V_R(\widehat{f},\pi^m,Q^m)$. Let

$$f_{\lambda_n}^m=\mathrm{argmin}_{f\in\mathcal{F}}(E[W_1^m\phi\{f(\boldsymbol{X})\}+W_{-1}^m\phi\{-f(\boldsymbol{X})\}]+\lambda_n\|f\|^2).$$

Then $\mathcal{A}(\lambda_n)$, defined in (5), is equal to

$$\lambda_n\|f_{\lambda_n}^m\|^2+\sum_{a=\pm 1} E\left[W_a^m\phi\{a\cdot f_{\lambda_n}^m(\boldsymbol{X})\}\right]-\sum_{a=\pm 1} E\left[W_a^m\phi\{a\cdot f^m(\boldsymbol{X})\}\right].$$

Recall that $W_a^m = W_a(Y, \boldsymbol{X}, A, \pi^m, Q^m)$ and $\widehat{W}_{ak} = W_a(Y, \boldsymbol{X}, A, \widehat{\pi}_k, \widehat{Q}_k)$. According to Proposition 3.1, we have,

$$
\begin{aligned}
& \psi[\{V_R(f^m, \pi^m, Q^m) - V_R(\widehat{f}, \pi^m, Q^m)\}/ \sup_{\boldsymbol{x}\in\mathbb{R}^p} c_m(\boldsymbol{x})] \\
\leq \quad & [R_\phi^m(\hat{f}) - R_\phi^{m^*}]/ \inf_{\boldsymbol{x}\in\mathbb{R}^p} c_m(\boldsymbol{x}) \\
\leq \quad & \frac{1}{K}\sum_{k=1}^K [R_\phi^m(\hat{f}_{n,k}^{\lambda_{n,k}}) - R_\phi^{m^*}]/ \inf_{\boldsymbol{x}\in\mathbb{R}^p} c_m(\boldsymbol{x})
\end{aligned}
$$

where the convexity of $R_\phi^m(\cdot)$ implies

$$
R_\phi^m(\hat{f}) = R_\phi^m\Big(\frac{1}{K}\sum_{k=1}^K \hat{f}_{n,k}^{\lambda_{n,k}}\Big) \leq \frac{1}{K}\sum_{k=1}^K R_\phi^m(\hat{f}_{n,k}^{\lambda_{n,k}}).
$$

Thus,

$$
\begin{aligned}
& R_\phi^m(\hat{f}_{n,k}^{\lambda_{n,k}}) - R_\phi^{m^*} \\
\leq \quad & E[W_1^m\phi\{\widehat{f}_{n,k}^{\lambda_{n,k}}(\boldsymbol{X})\}] - E[W_1^m\phi\{f^m(\boldsymbol{X})\}] + E[W_{-1}^m\phi\{-\widehat{f}_{n,k}^{\lambda_{n,k}}(\boldsymbol{X})\}] - E[W_{-1}^m\phi\{-f^m(\boldsymbol{X})\}] \\
\leq \quad & \lambda_n\|f_{\lambda_n}^m\|^2 + \sum_{a=\pm1}\Big\{E\left[W_a^m\phi\{a\cdot f_{\lambda_n}^m(\boldsymbol{X})\}\right] \\
& -E[W_a^m\phi\{a\cdot f^m(\boldsymbol{X})\}] + \lambda_n\|\widehat{f}_{n,k}^{\lambda_{n,k}}\|^2 + E[W_a^m\phi\{a\cdot \widehat{f}_{n,k}^{\lambda_{n,k}}(\boldsymbol{X})\}] \\
& - \left(\lambda_n\|f_{\lambda_n}^m\|^2 + E\left[W_a^m\phi\{a\cdot f_{\lambda_n}^m(\boldsymbol{X})\}\right]\right)\Big\} \\
= \quad & \mathcal{A}(\lambda_n) + \sum_a\Big[\lambda_n\|\widehat{f}_{n,k}^{\lambda_{n,k}}\|^2/2 + E[W_a\phi\{a\cdot\widehat{f}_{n,k}^{\lambda_{n,k}}(\boldsymbol{X})\}] - \lambda_n\|f_{\lambda_n}^m\|^2/2 - E[W_a\phi\{a\cdot f_{\lambda_n}^m(\boldsymbol{X})\}]\Big] \\
= \quad & \mathcal{A}(\lambda_n) + (I).
\end{aligned}
$$

We now bound (I) using empirical process theory. Let

$$
\mathcal{L}_f = \{\lambda_n\|f\|^2 + \sum_{a=\pm1}[W_a\phi\{a\cdot f(\boldsymbol{X})\} - W_a\phi\{a\cdot f_{\lambda_n}^m(\boldsymbol{X})\}] - \lambda_n\|f_{\lambda_n}^m\|^2, f\in\mathcal{B}(B_n)\},
$$

where $\mathcal{B}(B_n)$ is defined in Lemma A.2. By Lemma A.2, it holds that

$$
\|l_f\|_\infty \leq 2M^2 + 2\Big|\sum_a W_a\phi\{a\cdot f(\boldsymbol{X})\}\Big| = O(\lambda_n^{-1/2}),
$$

for any $f\in\mathcal{B}(B_n)$. It can be shown that $E(l_f^2)\leq c_n' E(l_f)$, where $c_n' = O(\lambda_n^{-1})$ following the arguments for proving Theorem 3.4 in Zhao et al. (2012). Suppose that l_f satisfies $\mathbb{P}_n(l_f)\leq\varepsilon/2$ and $E(l_f)\geq\varepsilon$ for some $\varepsilon>0$ to be chosen (note that $E(l_{f_{\lambda_n}^m}) = 0$). By the continuity of ϕ, there exists an $f' = t'f + (1-t')f_{\lambda_n}^m \in\mathcal{B}(B_n)$, which for $0\leq t'\leq 1$, has $E(l_{f'}) = \varepsilon$. In addition, by the convexity of ϕ,

$$
\begin{aligned}
\mathbb{P}_n{}^{(-k)}(l_{f'}) &\leq \mathbb{P}_n{}^{(-k)}\Big[t'\lambda_n\|f\|^2 + t'\sum_{a=\pm1}W_a\phi\{a\cdot f(\boldsymbol{X})\} - t'\lambda_n\|f_{\lambda_n}^m\|^2 - t'\sum_{a=\pm1}W_a\phi\{a\cdot f_{\lambda_n}^m(\boldsymbol{X})\}\Big] \\
&\leq t'\varepsilon/2 \leq \varepsilon/2.
\end{aligned}
$$

To apply Lemma A.1, we need to further verify that

$$\mathbb{P}_n^{(-k)}(l_{\widehat{f}_{n,k}^{\lambda_{n,k}}}) \le \varepsilon/2. \tag{4}$$

To show (4), note that

$$\begin{aligned}\mathbb{P}_n^{(-k)}(l_{\widehat{f}_{n,k}^{\lambda_{n,k}}}) &= \mathbb{P}_n^{(-k)}\Big[\lambda_n\|\widehat{f}_{n,k}^{\lambda_{n,k}}\|^2 + \sum_a W_a\phi\{a\cdot \widehat{f}_{n,k}^{\lambda_{n,k}}(\boldsymbol{X})\} - \lambda_n\|f_{\lambda_n}^m\|^2 - \sum_a W_a\phi\{a\cdot f_{\lambda_n}^m(\boldsymbol{X})\}\Big]\\ &= \mathbb{P}_n^{(-k)}\Big[\lambda_n\|\widehat{f}_{n,k}^{\lambda_{n,k}}\|^2 + \sum_a \widehat{W}_{ak}\phi\{a\cdot \widehat{f}_{n,k}^{\lambda_{n,k}}(\boldsymbol{X})\} - \lambda_n\|f_{\lambda_n}^m\|^2 - \sum_a \widehat{W}_{ak}\phi\{a\cdot f_{\lambda_n}^m(\boldsymbol{X})\}\Big]\\ &\quad - \mathbb{P}_n^{(-k)}\sum_a(\widehat{W}_{ak}-W_a)\phi\{a\cdot \widehat{f}_{n,k}^{\lambda_{n,k}}(\boldsymbol{X})\} + \mathbb{P}_n\sum_a(\widehat{W}_{ak}-W_a)\phi\{a\cdot f_{\lambda_n}^m(\boldsymbol{X})\}\\ &\le 0 + C(\lambda_n^{-1/2}n^{-(\alpha+\beta)} + n^{-(1/2+\min(\alpha,\beta))}\lambda_n^{-1/2}),\end{aligned}$$

where the last step hods by the definition of $\widehat{f}_{n,k}^{\lambda_{n,k}}$ and Lemma A.3. In the following, we use C to denote a generic constant, which could differ from line to line. Thus, provided $\varepsilon \ge C(\lambda_n^{-1/2}n^{-(\alpha+\beta)} + n^{-(1/2+\min(\alpha,\beta))}\lambda_n^{-1/2})$, (4) holds. Therefore, Lemma A.1 implies that with probability tending to one,

$$E\Big[\lambda_n\|\widehat{f}_{n,k}^{\lambda_{n,k}}\|^2 + \sum_{a=\pm1} W_a\phi\{a\cdot \widehat{f}_{n,k}^{\lambda_{n,k}}(\boldsymbol{X})\} - \lambda_n\|f_{\lambda_n}^m\|^2 - \sum_{a=\pm1} W_a\phi\{a\cdot f_{\lambda_n}^m(\boldsymbol{X})\}\Big] \le \varepsilon,$$

provided that

$$\varepsilon \ge \max\Big\{\varepsilon^*, \frac{Cc_n'K}{n}, \frac{CKB_n}{n}, C(\lambda_n^{-1/2}n^{-(\alpha+\beta)} + n^{-(1/2+\min(\alpha,\beta))}\lambda_n^{-1/2})\Big\},$$

where K is a constant, $B_n = O(\lambda_n^{-1/2})$, $c_n' = O(\lambda_n^{-1})$ and $\varepsilon^* \ge 12\xi_{\mathcal{L}_f}(\varepsilon^*)$. It remains to find ε^*.

Define $\mathcal{G}_f = \{E(l_f) - l_f : E(l_f) = \varepsilon, l_f \in \mathcal{L}_f\}$, then $E(g_f) = 0$ for any $g_f \in \mathcal{G}_f$. Let $Z = \sup_{g_f\in\mathcal{G}_f}\mathbb{P}_n g_f(\boldsymbol{X})$, then by definition of $\xi_{\mathcal{L}_f}$ (see Lemma A.1),

$$\xi_{\mathcal{L}_f}(\varepsilon) = E(Z) = E\Big\{\sup_{g_f\in\mathcal{G}_f}\mathbb{P}_n g_f(\boldsymbol{X})\Big\} = E\Big[\sup_{E(l_f^2)\le c_n'\varepsilon}\Big|E\{l_f(\boldsymbol{X})\} - \mathbb{P}_n l_f(\boldsymbol{X})\Big|\Big].$$

Because $f \in \mathcal{B}(B_n)$, there exists a constant depending on v, so that $\sup_P \log N(\epsilon, \mathcal{B}(B_n), L_2(P)) \le C_v(\epsilon/B_n)^{-v}$. It follows that

$$\begin{aligned}\log N\{\epsilon, \mathcal{L}_f, L_2(P)\} &\le \log N\{\epsilon, \mathcal{B}(B_n), L_2(P)\} + \log N\big\{\epsilon, \{\lambda_n\|f\|^2, f\in\mathcal{B}(B_n)\}, L_2(P)\big\}\\ &\le C\Big(\frac{\epsilon}{B_n}\Big)^{-v} + \log(M^2/\epsilon)\\ &\le CB_n^v\epsilon^{-v}.\end{aligned}$$

Hence, $E(Z)$ is bounded above by

$$C\max\Big\{n^{-\frac{2}{v+2}}\lambda_n^{-\frac{v}{2+v}}, n^{-\frac{1}{2}}\lambda_n^{-\frac{v}{4}}\varepsilon^{\frac{2-v}{4}}\Big\}, \tag{5}$$

by Proposition 5.5 in Steinwart and Scovel (2007). Consequently, it suffices to choose

$$\varepsilon^* = Cn^{-\frac{2}{v+2}}\lambda_n^{-\frac{v}{v+2}},$$

and by solving $\varepsilon \geq 12\xi_{\mathcal{L}_f}(\varepsilon)$ for ε^*, i.e., $\varepsilon \geq 12E(Z)$, where $E(Z)$ can be replaced with (5). Therefore,

$$\varepsilon \geq \max\left\{Cn^{-\frac{2}{v+2}}\lambda_n^{-\frac{v}{v+2}}, \frac{Cc_n' K}{n}, \frac{CKB_n}{n}, C(\lambda_n^{-1/2}n^{-(\alpha+\beta)} + n^{-(1/2+\min(\alpha,\beta))}\lambda_n^{-1/2})\right\}.$$

This completes the proof.

Lemma A.1. [Lemma 6 from Bartlett et al. (2006)] Consider a class $\mathcal{F}$ of functions $f : \mathcal{X} \to \mathbb{R}$ with $\sup_{f\in\mathcal{F}} \|f\|_\infty \leq B$. Let P be a probability distribution on $\mathcal{X}$, and suppose that there exist $c \geq 1$ and $0 < \beta \leq 1$ such that, for all $f \in \mathcal{F}$, $Ef^2(X) \leq c(Ef)^\beta$. Fix $0 < \alpha, \epsilon < 1$. Suppose that if some $f \in \mathcal{F}$ has $\mathbb{P}_n f \leq \alpha\epsilon$ and $Ef \geq \epsilon$, then some $f' \in \mathcal{F}$ has $\mathbb{P}_n f' \leq \alpha\epsilon$ and $Ef' = \epsilon$. Then with probability at least $1 - e^{-x}$, any $f \in \mathcal{F}$ satisfies $\mathbb{P}_n f \leq \alpha\epsilon \Rightarrow Ef \leq \epsilon$, provided that

$$\epsilon \geq \max\left\{\epsilon^*, \left(\frac{9cKx}{(1-\alpha)^2 n}\right)^{1/(2-\beta)}, \frac{4KBx}{(1-\alpha)n}\right\},$$

where K is an absolute constant and $\epsilon^* \geq 6\xi_{\mathcal{F}}(\epsilon^*)/(1-\alpha)$, with $\xi_{\mathcal{F}}(\epsilon) = E\sup[E(f) - \mathbb{P}_n(f) : f \in \mathcal{F}, E(f) = \epsilon]$.

Lemma A.2. Under the same conditions as in Theorem 3.1, we have for $a = 1$ or -1, $|\phi(a \cdot \hat{f}_{n,k}^{\lambda_{n,k}})| \lesssim \lambda_n^{-1/2}$ and $|\phi(a \cdot f_{\lambda_n}^m)| \lesssim \lambda_n^{-1/2}$.

Proof According to Assumption 2, $|\widehat{W}_{ak}|$ can be bounded above by a constant. We then obtain a trivial bound for $\|\widehat{f}_{n,k}^{\lambda_{n,k}}\|$, as $\mathbb{P}_n{}^{(-k)}[\sum_{a=\pm1}\widehat{W}_{ak}\phi\{a \cdot \widehat{f}_{n,k}^{\lambda_{n,k}}(\boldsymbol{X})\} + \lambda_n\|\widehat{f}_{n,k}^{\lambda_{n,k}}\|^2] \leq \mathbb{P}_n{}^{(-k)}[\sum_{a=\pm1}\widehat{W}_{ak}\phi(0)]$, then $\|\widehat{f}_{n,k}^{\lambda_{n,k}}\| \leq \left\{\lambda_n^{-1}\mathbb{P}_n(\sum_{a=\pm1}\widehat{W}_{ak})\right\}^{1/2} \leq M\lambda_n^{-1/2}$, where M^2 is a constant bounding the empirical average. Similarly,

$$\lambda_n\|f_{\lambda_n}^m\|^2 \leq \inf_{f\in\mathcal{F}} \lambda_n\|f\|^2 + E\Big[\sum_{a=\pm1} W_a^m\phi\{a \cdot f(\boldsymbol{X})\}\Big] \leq \sum_{a=\pm1} E\left[W_a^m\phi(0)\right],$$

so that $\|f_{\lambda_n}^m\| \leq M\lambda_n^{-1/2}$. Set $B_n = M\lambda_n^{-1/2}$ and let $\mathcal{B}(B_n)$ denote a ball of radius B_n in $\mathcal{F}$ that is centered at zero. For every $f \in \mathcal{B}(B_n)$, we have $|\phi(a \cdot f)| \leq L\|f\| + \phi(0) \leq ML\lambda_n^{-1/2} + \phi(0)$, where L is the Lipschitz constant of the convex loss. This completes the proof. ■

Lemma A.3. Under the same conditions as in Theorem 3.1, we have for $a = 1$ or -1,

$$|\mathbb{P}_n{}^{(-k)}(\widehat{W}_{ak} - W_a)\phi(af_{\lambda_n}^m(\boldsymbol{X}))| = O_p(\lambda_n^{-1/2}n^{-(\alpha+\beta)} + n^{-(1/2+\min(\alpha,\beta))}\lambda_n^{-1/2}), \quad (6)$$

$$|\mathbb{P}_n{}^{(-k)}(\widehat{W}_{ak} - W_a)\phi(a\hat{f}_{n,k}^{\lambda_{n,k}}(\boldsymbol{X}))| = O_p(\lambda_n^{-1/2}n^{-(\alpha+\beta)} + n^{-(1/2+\min(\alpha,\beta))}\lambda_n^{-1/2}). \quad (7)$$

Proof For simplicity, we only consider $a = 1$. For notational simplicity, we use $\hat{Q}$, $\hat{\pi}$ and $\hat{f}$ to denote $\hat{Q}_k$, $\hat{\pi}_k$ and $\hat{f}_{n,k}^{\lambda_{n,k}}$. For (6),

$$
\begin{aligned}
&\Big|\mathbb{P}_n{}^{(-k)}(\widehat{W}_1 - W_1)\phi(\hat{f}(\boldsymbol{X}))\Big| \\
&= \Big|\mathbb{P}_n{}^{(-k)}\Big[\frac{YI\{A=1\}}{\hat{\pi}(1;\boldsymbol{X})} - \frac{I\{A=1\}-\hat{\pi}(1;\boldsymbol{X})}{\hat{\pi}(1;\boldsymbol{X})}\hat{Q}(\boldsymbol{X},1) - \frac{YI\{A=1\}}{\pi(1;\boldsymbol{X})} \\
&\qquad - \frac{I\{A=1\}-\pi(1;\boldsymbol{X})}{\pi(1;\boldsymbol{X})}Q(\boldsymbol{X},1)\Big]\phi(\hat{f}(\boldsymbol{X}))\Big| \\
&\le \Big|\mathbb{P}_n{}^{(-k)}\Big(\frac{I\{A=1\}}{\hat{\pi}(1;\boldsymbol{X})} - \frac{I\{A=1\}}{\pi(1;\boldsymbol{X})}\Big)\Big(\hat{Q}(\boldsymbol{X},1) - Q(\boldsymbol{X},1)\Big)\phi(\hat{f}(\boldsymbol{X}))\Big| \\
&\qquad + \Big|\mathbb{P}_n{}^{(-k)}\Big(\frac{I\{A=1\}}{\hat{\pi}(1;\boldsymbol{X})} - \frac{I\{A=1\}}{\pi(1;\boldsymbol{X})}\Big)\Big(Y - Q(\boldsymbol{X},1)\Big)\phi(\hat{f}(\boldsymbol{X}))\Big| \\
&\qquad + \Big|\mathbb{P}_n{}^{(-k)}\Big(\frac{I\{A=1\}}{\pi(1;\boldsymbol{X})} - 1\Big)\Big(Q(\boldsymbol{X},1) - \hat{Q}(\boldsymbol{X},1)\Big)\phi(\hat{f}(\boldsymbol{X}))\Big| \\
&= (I) + (II) + (III).
\end{aligned}
$$

We first consider (III). Let

$$\mathcal{F}_{Q,1} = \Big\{\Big(\frac{I\{A=1\}}{\pi(1;\boldsymbol{X})} - 1\Big)\Big(Q(\boldsymbol{X},1) - \hat{Q}(\boldsymbol{X},1)\Big)\phi(f(\boldsymbol{X})), f \in \mathcal{B}(B_n)\Big\}.$$

Since $\boldsymbol{X}$ and $\hat{Q}$ are independent by sample splitting, we have $E(g) = 0$ for any $g \in \mathcal{F}_{Q,1}$. Then

$$(III) \lesssim n^{-1/2} E \sup_{g\in\mathcal{F}_{Q,1}} |\mathbb{G}_n g|.$$

The envelop function is given by $F_{Q,1} = CM|(I\{A=1\}/\pi(1;\boldsymbol{x})-1)(Q(\boldsymbol{X},1)-\hat{Q}(\boldsymbol{X},1))|\lambda_n^{-1/2}$. Therefore, $\|F_{Q,1}\|_{P,2} = Cn^{-\beta}\lambda_n^{-1/2}$ by the L_2 convergence of $\hat{Q}_k$. By our entropy assumption, $\sup_P \log N(\epsilon, \mathcal{B}(B_n), L_2(P)) \le C(\epsilon/B_n)^{-v}$. By the Lipschitz propensity of $\pi(\cdot)$, we have $\sup_P \log N(L\epsilon, \{\phi(f) : f \in \mathcal{B}(B_n)\}, L_2(P)) \le C(\epsilon/B_n)^{-v}$. This further implies

$$\sup_P \log N(\epsilon\|F_{Q,1}\|_{P,2}, \mathcal{F}_{Q,1}, L_2(P)) \le C\epsilon^{-v}.$$

Thus,

$$J(1, \mathcal{F}_{Q,1}, L_2) = \int_0^1 \sqrt{\log\sup_P N(\epsilon\|F_{Q,1}\|_{P,2}, \mathcal{F}_{Q,1}, L_2(P))}d\epsilon \lesssim 1.$$

Thus, by the maximal inequality in Lemma 19.38 in Van der Vaart (2000), we obtain that

$$(III) \lesssim n^{-1/2}n^{-\beta}\lambda_n^{-1/2}$$

We can similarly show that

$$(II) \lesssim n^{-1/2}n^{-\alpha}\lambda_n^{-1/2}$$

Finally, we consider (I),

$$
\begin{aligned}
(I) &\le \Big\{\mathbb{P}_n{}^{(-k)}\Big(\frac{I\{A=1\}}{\hat\pi(1;\boldsymbol{X})}-\frac{I\{A=1\}}{\pi(1;\boldsymbol{X})}\Big)^2\Big\}^{1/2}\cdot\Big\{\mathbb{P}_n{}^{(-k)}\Big(\hat Q(\boldsymbol{X},1)-Q(\boldsymbol{X},1)\Big)^2\phi^2(\hat f(\boldsymbol{X}))\Big\}^{1/2}\\
&\lesssim \Big\{\mathbb{P}_n{}^{(-k)}\Big(\hat\pi(1;\boldsymbol{X})-\pi(1;\boldsymbol{X})\Big)^2\Big\}^{1/2}\cdot\Big\{\mathbb{P}_n{}^{(-k)}\Big(\hat Q(\boldsymbol{X},1)-Q(\boldsymbol{X},1)\Big)^2\Big\}^{1/2}\lambda_n^{-1/2}\\
&\lesssim n^{-(\alpha+\beta)}\lambda_n^{-1/2},
\end{aligned}
$$

since $\mathbb{P}_n{}^{(-k)}\Big(\hat Q(\boldsymbol{X},1)-Q(\boldsymbol{X},1)\Big)^2\lesssim E\Big(\hat Q(\boldsymbol{X},1)-Q(\boldsymbol{X},1)\Big)^2=E\|\hat Q(\boldsymbol{X},1)-Q(\boldsymbol{X},1)\|_{P,2}^2\le Cn^{-2\beta}$ by the independence between $\boldsymbol{X}$ and $\hat Q$. Combining the bounds for $(I),(II),(III)$, we obtain (7). The first result (6) is also implied by the above proof. ■

Web Appendix E: Proof of Corollary 3.1

Provided that

$$
\begin{aligned}
V^*-\bar V &= \frac{1}{K}\sum_{k=1}^{K}[V^*-\hat V_{(-k)}(\hat f_{n,k}^{\lambda_{n,k}})]\\
&= \frac{1}{K}\sum_{k=1}^{K}[V^*-V(\hat f_{n,k}^{\lambda_{n,k}})+V(\hat f_{n,k}^{\lambda_{n,k}})-\hat V_{(-k)}(\hat f_{n,k}^{\lambda_{n,k}})],
\end{aligned}
$$

we only need to derive the bound for the rate of convergence of $V(\hat f_{n,k}^{\lambda_{n,k}})-\hat V_{(-k)}(\hat f_{n,k}^{\lambda_{n,k}})$.

$$
\begin{aligned}
&V(\hat f_{n,k}^{\lambda_{n,k}})-\hat V_{(-k)}(\hat f_{n,k}^{\lambda_{n,k}})\\
=\;& \mathbb{P}_n{}^{(-k)}\Big[(\widehat W_a-W_a)I\{a=\operatorname{sgn}(\widehat{f}_{n,k}^{\lambda_{n,k}})\}\Big]+\sum_{a=\pm1}(\mathbb{P}_n{}^{(-k)}-\mathbb{P})[W_aI\{a=\operatorname{sgn}(\widehat{f}_{n,k}^{\lambda_{n,k}})\}]\\
\le\;& \Bigg|\sum_{a=\pm1}(\mathbb{P}_n{}^{(-k)}-\mathbb{P})[W_aI\{a=\operatorname{sgn}(\widehat{f}_n^{\lambda_n})\}]\Bigg|+O_p(\lambda_n^{-1/2}n^{-(\alpha+\beta)}+n^{-(1/2+\min(\alpha,\beta))}\lambda_n^{-1/2}),
\end{aligned}
$$

where the last step is from Lemma A.3, because $\hat Q_k,\hat\pi_k$ and $\mathbb{P}_n{}^{(-k)}$ are independent. We consider $a=1$, and the case with $a=-1$ follows similarly. Since the norm of $W_aI\{a=\operatorname{sgn}(\widehat{f}_n^{\lambda_n})\}$ is bounded, the class $\mathcal{F}_1=\{W_1I[1=\operatorname{sgn}\{f(\boldsymbol{X})\}],f\in\mathcal{B}(B_n)\}$ is contained in a Donsker class. Then,

$$
\Big|(\mathbb{P}_n-\mathbb{P})[W_1I\{1=\operatorname{sgn}(\widehat{f}_n^{\lambda_n})\}]\Big|\le n^{-1/2}\sup_{g\in\mathcal{F}_1}|\mathbb{G}_ng|=O_p(n^{-1/2}).
$$

The first result thus follows. Finally, as seen in the proof of Theorem 3.1, $\frac{1}{K}\sum_{k=1}^{K}[V^*-V(\hat f_{n,k}^{\lambda_{n,k}})]$ has the desired convergence rate. This completes the proof.

Web Appendix F: Additional simulations studies

F.1 Comparing convex relaxations

We also compared the performance across different surrogate loss functions. We considered logistic loss, exponential loss, squared hinge loss, and hinge loss applied with modeling choices CC, CI, IC, and II and sample sizes 100, 150, 200, 250, 300, 350, 400, 500, 600, 700, 800, 1000, 1200 and 1600. Figures 1 and 2 display the results which show that smooth losses performed similarly, all exhibiting better performances when the regression model is correct; however, hinge loss has a more robust performance when the regression model is incorrect.

Web Figure 1: Mean values using different surrogate loss functions under EARL in Scenario 1

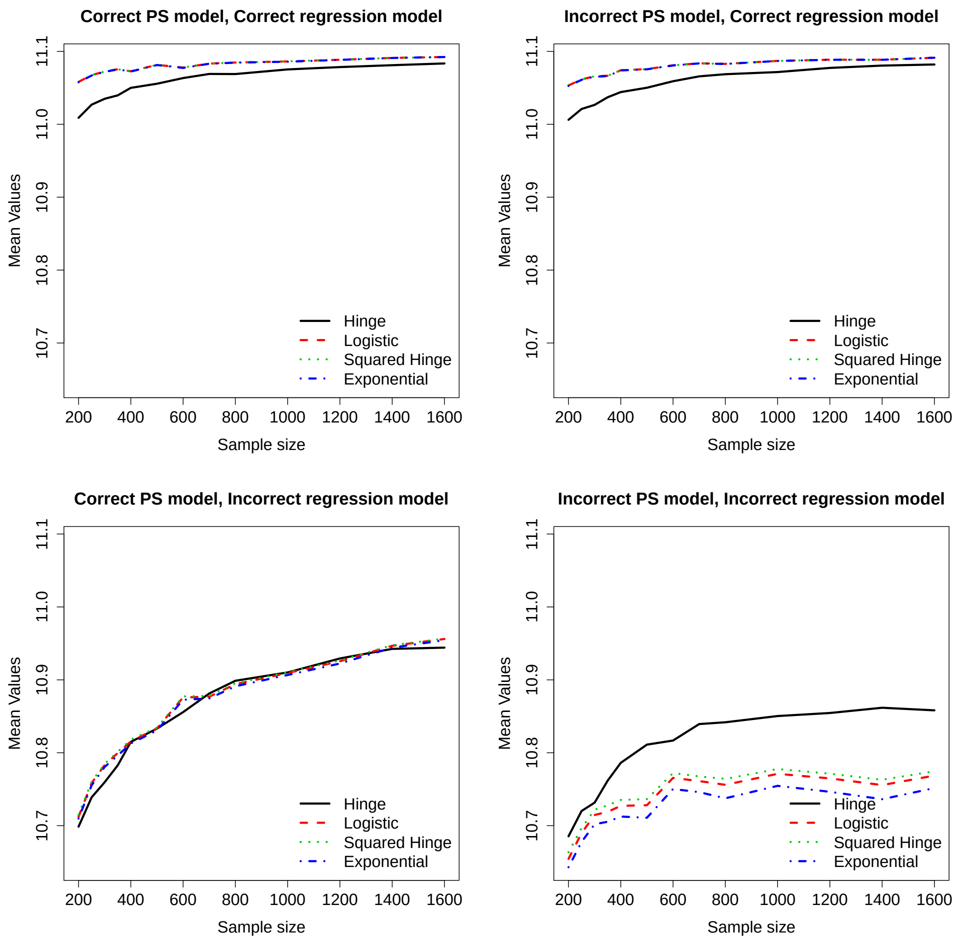

Web Figure 2: Mean values using different surrogate loss functions under EARL in Scenario 2

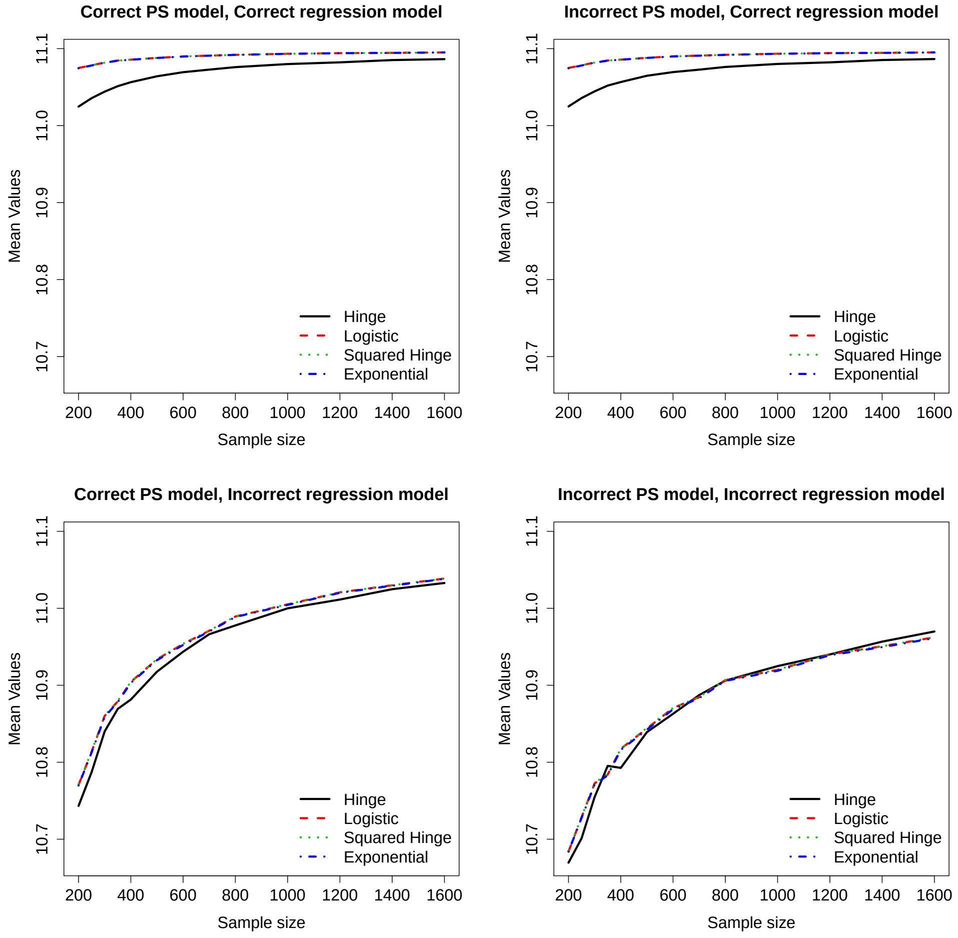

F.2 Parametric vs. nonparametric working models

We also conducted a set of simulation experiments to investigate the role of parametric and nonparametric models for the propensity score and outcome regression. We consider the following modeling choices.

CN. A correctly specified logistic regression model for $\pi(A; \boldsymbol{X})$ with predictors X_1, X_2 and X_1X_2, and a nonparametric estimator for $Q(\boldsymbol{X}, A)$ fit using a random forest.

IN. An incorrectly specified logistic regression model for $\pi(A; \boldsymbol{X})$ with predictors $\boldsymbol{X}$, and a nonparametric estimator for $Q(\boldsymbol{X}, A)$ fit using a random forest.

NC. A nonparametric estimator for $\pi(A; \boldsymbol{X})$ fit using a random forest, and a correctly specified linear model for $Q(\boldsymbol{X}, A)$ with predictors $\boldsymbol{X}, \boldsymbol{X}^2, A, X_1A$ and X_1^2A.

NI. A nonparametric estimator for $\pi(A; \boldsymbol{X})$ fit using a random forest, and an incorrectly specified linear model for $Q(\boldsymbol{X}, A)$ with predictors $\boldsymbol{X}, A, \boldsymbol{X}A$.

NN. Nonparametric estimators for both $\pi(A; \boldsymbol{X})$ and $Q(\boldsymbol{X}, A)$ each fit using random forests.

As in the previous section, logistic loss was used and sample sizes considered were 200, 400, 800 and 1600. The results for Scenarios 1 and 2 are shown in Figures 3 and 4. In the examples considered, using nonparametric working models for propensity scores could improve results over parametric models.

Web Appendix G: Application: National Supported Work (NSW)

The National Supported Work (NSW) Demonstration was a temporary employment program implemented in mid-1970s. The program was designed to prepare disadvantaged workers for the labor market by providing them with work experience and counseling. NSW was designed as a randomized clinical trial in which subjects were randomly assigned to the NSW-exposed group and the unexposed group. LaLonde (1986) created a composite observational dataset by taking subjects in the NSW-exposed group and the nonexperimental control group from other sources. The NSW data, including the mixed NSW data, has been extensively analyzed to evaluate the impact of the NSW program on post-intervention income levels (LaLonde, 1986; Dehejia and Wahba, 1999). We now use the composite NSW data, in which treatments were not randomized, to illustrate the EARL method for identifying the optimal treatment rules. The data set is available in the `R` package `MatchIt` (Ho et al., 2011).

There are 614 observations, with 185 subjects in the NSW-exposed group and 429 in the control group. We used the change in reported earnings from 1975 to 1978 as the primary outcome. We used eight baseline covariates, including age, schooling years, 1974 earnings (on log scale), race (black or white), ethnicity (Hispanic origin or not), marital status (married or not), and degree (has a high school degree or not). We used linear and logistic regression models to estimate the Q-function and propensity score. For comparison, we also estimated an optimal treatment rule using L_1-PLS and OWL. As in the previous example, inverse probability weighting was used to assess the quality of the estimated rule. The estimated treatment rule when using the EARL estimator with logistic loss assigned 397 subjects to the NSW group, and 217 patients to the unexposed group. This rule indicated that subjects who are younger, less educated, and have a lower income in 1974 are more likely to benefit from the NSW program. The estimated value of this rule was 5884.5. In comparison, the treatment rule estimated using L_1-PLS recommended 244 subjects to the NSW group and had a lower estimated value of 5227.0. The treatment rule estimated using OWL with a logistic loss led to an estimated value of 5110.9.

Web Appendix H: Example code for implementing EARL method

Here we provide the codes for running simulation Scenario 2 using correct outcome regression and propensity score models. More details can be found in help file of R package 'DynTxRegime'.

```
library(DynTxRegime)

## Generate training data
n = 500
p = 10
X = matrix(rnorm(n*p), n, p)
pi = 0.5*X[, 1] - 0.5
propensity = exp(pi) / (1 + exp(pi))
A = 2*rbinom(n, 1, propensity) - 1
mX = apply(X[ , 1:10]^2, 1, sum)+apply(X[ ,1:10], 1, sum)
cX = -X[ , 1] + X[ , 2]  - .1
R = mX + A*cX  + rnorm(n, 1)

## Generate validation data

testn = 10000
testX  = matrix(rnorm(testn * p), testn, p)
testmX = apply(testX[ , 1:10]^2, 1, sum)+apply(testX[ ,1:10], 1, sum)
testcX =  -testX[ , 1]  +  testX[ , 2]  - .1


## Prepare for the data

a =  as.numeric(A > 0)
data = data.frame(1, X, X^2, X[ , 1] * X[ , 2], a, R)
names(data) = c("x0", paste0("x", 1:(2 * p+1)), "a", "R")
newData = data.frame(1, testX, testX^2, testX[ , 1]*testX[ , 2])
names(newData) = c("x0", paste0("x", 1 : (2 * p+1)))

## The following example uses correct outcome regression and
## correct propensity score models.

##  Outcome regression model consists of main effect component and contrast component.
##  Create modeling object for main effect component, which contains X^2 and X terms.
mod1 = paste0("x", 1 : (2 * p))
mod1 = paste(mod1, collapse=" + ")
mod_main_correct = paste("model = ~", mod1)
mod_main_correct = as.formula(mod_main_correct)
expec.mainCorrect = buildModelObj(model =  mod_main_correct,
```

```
                                  solver.method = 'lm')


##   Create modeling object for contrast component, which contains X1 and X2
expec.contCorrect  = buildModelObj(model =  ~   x1 + x2,
                                   solver.method = 'lm')

##  Propensity score model includes x1 as covariate.
##  Create modeling object for propensity score.
propenCorrect = buildModelObj(model =  ~ x1,
                              solver.method = 'glm',
                              solver.args = list('family'='binomial'),
                              predict.method = 'predict.glm',
                              predict.args = list(type='response'))

##  Specify the form of regimes.
##  Here, we use regime of the form ~ x0 + x1 + ... + x10.
regime1 = c(paste0("x", 0:p))
regime1 = paste(regime1, collapse=" + ")
regime1 = paste("model = ~", regime1)
regime1 = as.formula(regime1)

##  EARL with both correct models.
earlRes = earl(moPropen = propenCorrect, moMain = expec.mainCorrect,
               moCont = expec.contCorrect,
               data = data, response = data$R,
               txName = 'a', surrogate = 'logit',
               regime = regime1, lambdas=2^seq(-5,5,1),
               cvFolds = 5, verbose = FALSE)



## Parameter estimates for decision function
regimeCoef(earlRes)

## Show main results of method
show(earlRes)

##  Show summary results of method
summary(earlRes)

##  Estimated optimal treatment for new data
optTx(earlRes, newdata)
```

References

Peter L Bartlett, Michael I Jordan, and Jon D McAuliffe. Convexity, classification, and risk bounds. *J. of American Statistical Association*, 101(473):138–156, 2006.

Rajeev H Dehejia and Sadek Wahba. Causal effects in nonexperimental studies: Reevaluating the evaluation of training programs. *Journal of the American statistical Association*, 94(448):1053–1062, 1999.

Daniel E Ho, Kosuke Imai, Gary King, and Elizabeth A Stuart. Matchit: nonparametric preprocessing for parametric causal inference. *Journal of Statistical Software*, 42(8), 2011.

Robert J LaLonde. Evaluating the econometric evaluations of training programs with experimental data. *The American Economic Review*, pages 604–620, 1986.

Ingo Steinwart and Clint Scovel. Fast rates for support vector machines using gaussian kernels. *The Annals of Statistics*, 35:575–607, 2007.

Aad W Van der Vaart. Asymptotic statistics (cambridge series in statistical and probabilistic mathematics). 2000.

Y. Q. Zhao, Donglin Zeng, A. John Rush, and Michael R. Kosorok. Estimating individualized treatment rules using outcome weighted learning. *Journal of American Statistical Association*, 107:1106–1118, 2012.

Web Figure 3: Boxplots for Scenario 1 results under EARL with different working models using logistic loss.

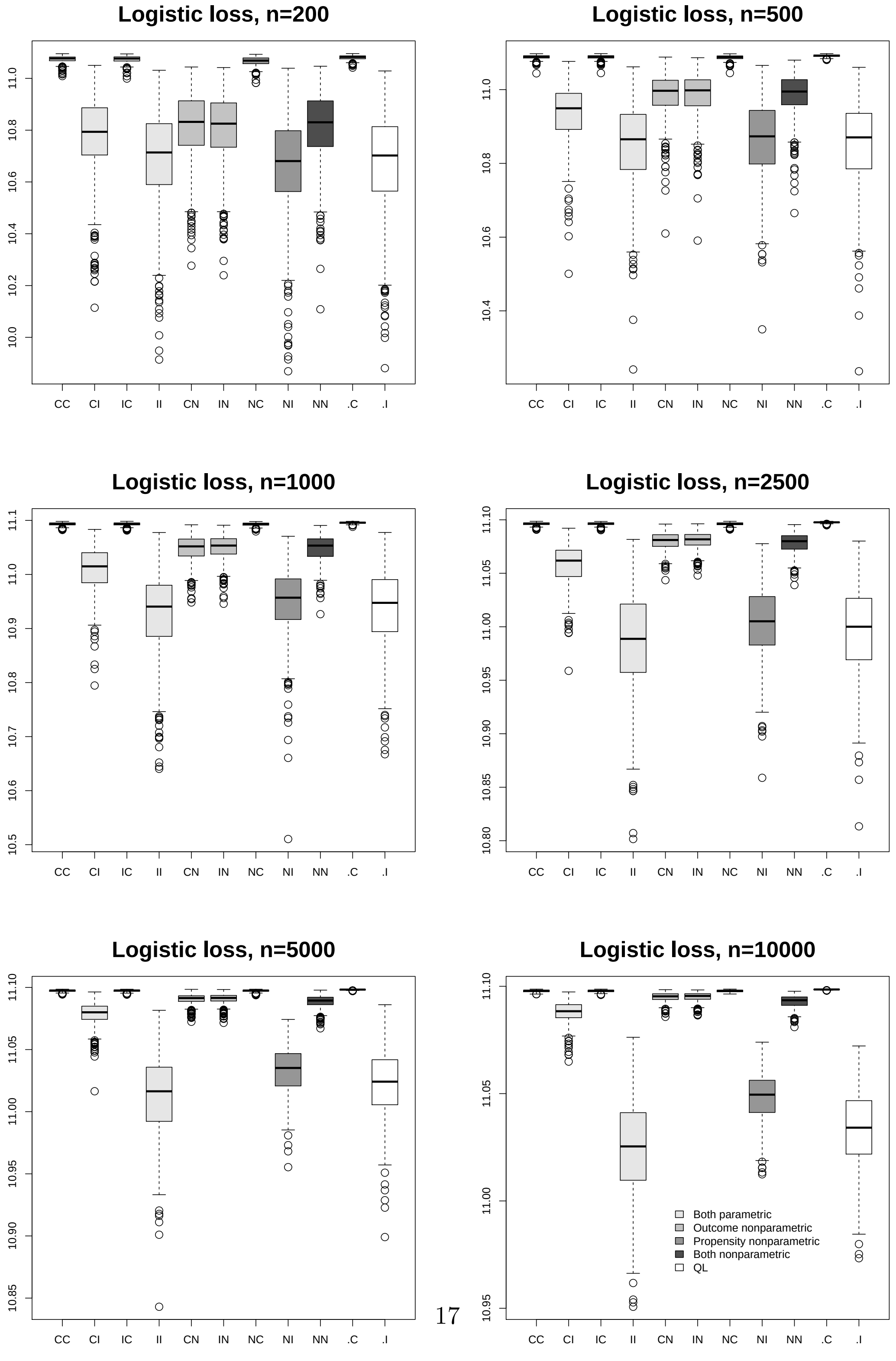

Web Figure 4: Boxplots for Scenario 2 results under EARL with different working models using logistic loss.

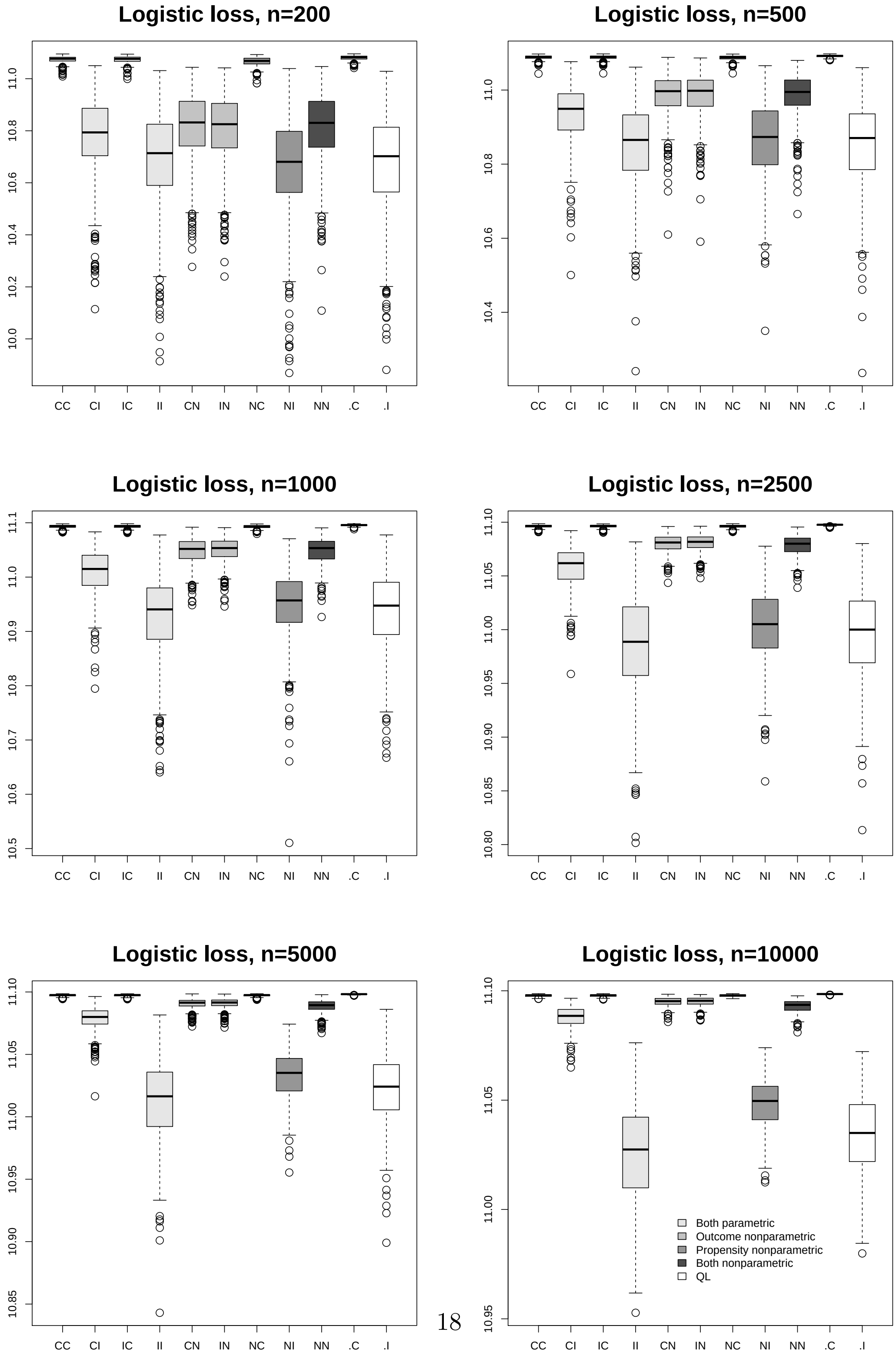

Supplement: Supplementary material [file NIHMS1040665-supplement-Supplementary_material.pdf]
